# Trabecular meshwork cellular mis-trafficking of near-native and premature stop variants in glaucoma-relevant myocilin

**DOI:** 10.1242/dmm.052821

**Published:** 2026-10-30

**Authors:** Kamisha R. Hill, Hailee F. Scelsi, Hannah A. Youngblood, Jennifer A. Faralli, Elijah T. Dunn, Tatsuo Itakura, M. Elizabeth Fini, Donna M. Peters, Raquel L. Lieberman

**Affiliations:** ^1^ School of Chemistry and Biochemistry, Georgia Institute of Technology, Atlanta, GA 30332, USA; ^2^ Department of Pathology and Laboratory Medicine, University of Wisconsin, Madison, WI 53705, USA; ^3^ USC Institute for Genetic Medicine, Keck School of Medicine of USC, University of Southern California, Los Angeles, CA 90033, USA; ^4^ New England Eye Center, Tufts Medical Center; Department of Ophthalmology, School of Medicine and Tufts Graduate School of Biomedical Sciences, Tufts University, Boston, MA 02111, USA

**Keywords:** Glaucoma, Proteostasis, ER-associated degradation, Autophagy, Genetic disease

## Abstract

Mutations in the myocilin olfactomedin (OLF) domain can cause vision loss in glaucoma by inducing protein misfolding in trabecular meshwork cells. However, not all mutations are causal for the disease. Here, we compare the cellular behaviors of two variants with uncertain contribution to glaucoma – namely, the missense variant A427T and premature stop variant Q368X – to those of wild type (WT) and disease variant Y437H. When expressed in an inducible immortalized trabecular meshwork cell line, inhibition of the proteasome rerouted WT myocilin from endoplasmic reticulum-associated degradation to autophagy. By contrast, neither Y437H, A427T nor Q368X could be rerouted for degradation. Conversely, inhibition of autophagy caused accumulation of WT myocilin and did not promote clearance of any tested mutant myocilins. Although the Q368X OLF mutant is misfolded, the crystal structure of the A427T OLF mutant showed modest perturbations that were largely confined to the mutation site. Our study demonstrates that a full range of misfolded states of mutant myocilin can present challenges for protein quality control clearance pathways and expands the range of defects known to impair mutant myocilin clearance.

## Teaser

**Summary:** Rare mutations in myocilin can cause glaucoma, and Q368X and A427T are of uncertain pathogenicity. In a new cellular model, Q368X and A427T exhibit defects similar to disease variant Y437H.

## INTRODUCTION

Missense mutations in the gene encoding myocilin (*MYOC*; UniProt accession Q99972) cause a subtype of glaucoma with a Mendelian inheritance pattern. Myocilin glaucoma accounts for 3-4% of all primary open angle glaucoma (POAG), the most common overall glaucoma subtype, and impacts ∼3 million children and young adults (Fingert et al., 1999; Resch and Fautsch, 2009; Stone et al., 1997). The primary and causal risk factor for glaucoma, elevated intraocular pressure (IOP), is brought about by trabecular meshwork (TM) dysfunction in the anterior segment of the eye (Gabelt and Kaufman, 2005). Wild-type (WT) myocilin (myocilin^WT^) is secreted from cells in the TM tissue that maintains IOP by promoting drainage of aqueous humor, a nourishing fluid in the anterior eye.

By contrast, disease-associated myocilin mutants aggregate in the endoplasmic reticulum (ER), causing a cytotoxic gain of function (Gould et al., 2006; Jacobson et al., 2001; Joe et al., 2003; Liu and Vollrath, 2004; Yam et al., 2007). Prior studies confirmed that mutant myocilin is not degraded and that the proteasomal pathway is involved in degrading myocilin (Qiu et al., 2014). More recently, using a HEK293T cellular model, our laboratory showed involvement of the ER chaperone Grp94 (also known as HSP90B1) in a dominant-negative mechanism that prevented efficient retrotranslocation of the severe mutant I477N for ER-associated degradation (ERAD); siRNA knockdown or selective inhibition of Grp94 with small molecules allows for degradation of disease-causing myocilins by autophagy (Huard et al., 2018; Stothert et al., 2014; Suntharalingam et al., 2012). Thus, myocilin glaucoma falls under the umbrella of proteostasis diseases. TM cells are long lived and quiescent (Liton et al., 2005) and, as such, are likely hardwired to avoid programmed cell death (Wang, 1995). The efficiency of the cellular proteostasis machinery declines with age, resulting in the accumulation of protein aggregates that lead to disease (Hipp et al., 2019). Proteostasis decline and disease onset are accelerated in cases of mutations in disease-associated genes (Bershtein et al., 2013; Santra et al., 2019; Wang and Moult, 2001).

Myocilin is a large multidomain protein, yet >90% of the known mutations are clustered within its C-terminal 30 kDa olfactomedin (OLF) domain, a five-bladed β-propeller spanning amino acids 245-504 (Donegan et al., 2015). Many different rare pathogenic mutations in OLF are found in populations around the world (Sun et al., 2022). In the clinic, pathogenic myocilin mutations are characterized by an autosomal dominant inheritance pattern with high penetrance, high IOP, specific optic nerve deformities and age at diagnosis <40 years (Gordon et al., 2002). In the laboratory, pathogenic mutations exhibit decreased protein stability, increased aggregation in the ER and multiple hallmarks of amyloid (Burns et al., 2010, 2011; Hill et al., 2014; Lieberman and Ma, 2021; Orwig et al., 2012). Not all myocilin mutations are pathogenic, however. Benign polymorphisms, usually identified in population-based genomic screening, are often indistinguishable from myocilin^WT^ in structure, stability and cellular behavior (Scelsi et al., 2023). These mutations, annotated as likely benign, are still rare, often with an allele frequency of ∼1×10^−6^ in the Genome Aggregation Database (gnomAD) database (Chen et al., 2024).

Here, we focus on two myocilin mutants of uncertain pathogenicity, missense variant A427T (myocilin^A427T^) and premature stop variant Q368X (myocilin^Q368X^). We show that, as recombinantly expressed and purified OLF domains, Q368X (OLF^Q368X^) is misfolded, whereas A427T (OLF^A427T^) exhibits modest structural perturbations that are largely confined to the mutation site. By using an inducible immortalized trabecular meshwork (TM-1) cell line with inducible myocilin, we compared the cellular behavior of myocilin^A427T^ and myocilin^Q368X^ to that of myocilin^WT^ and disease variant Y437H (myocilin^Y437H^). We show that whereas myocilin^WT^ can be rerouted to autophagy upon proteasomal inhibition, neither myocilin^Y437H^, myocilin^Q368X^ nor myocilin^A427T^ is degraded under similar conditions. Conversely, inhibition of autophagic clearance decreases cellular degradation of WT myocilin and all three variants tested. Thus, both extremes of misfolded states of myocilin can present challenges for quality control clearance pathways in TM cells. Our study expands the range of protein defects known to impair mutant myocilin clearance and supports a therapeutic strategy aimed at overcoming these deficiencies in cellular clearance.

## RESULTS

### The genetics of myocilin^Q368X^ and myocilin^A427T^ differ from that of canonical disease-causing myocilin mutations

Myocilin^Q368X^, which introduces a premature stop codon in the middle of the OLF domain sequence, is the third most prevalent myocilin missense variant currently documented (Fingert et al., 1999; Gong et al., 2004). Q368X is listed as pathogenic by the ClinVar consortium (Landrum et al., 2018); however, Q368X has been identified in the general population (Aldred et al., 2004; Fingert et al., 1999) and in POAG cases (Allingham et al., 1998; Ennis et al., 2010). Because Q368X has variable penetrance, it may be better described as a disease modifier (Han et al., 2019; Nag et al., 2017). Namely, when combined with other genetic and environmental risk factors, Q368X confers an increased risk for the development of disease (Liu and He, 2007; Wiggs et al., 2013; Young et al., 2012). In gnomAD, a database that does not include clinical data, the total allele frequency of Q368X is 1.16×10^−3^, with the highest prevalence in Caucasian populations (allele frequency 1.43×10^−3^) (Table S1).

In contrast, myocilin^A427T^ is a rare myocilin missense variant with some characteristics that are reminiscent of disease variants. A427T is considered of uncertain pathogenicity by the ClinVar consortium (Landrum et al., 2018). A427T was initially identified in a sporadic case of POAG diagnosed at age 73, but the mutation was later found in other family members (Faucher et al., 2002). Unlike bona fide pathogenic variants, however, other family members carrying the mutation around the same age as the proband were diagnosed with other types of glaucoma, and several other younger family members were asymptomatic despite carrying the mutation. More sporadic POAG cases having myocilin^A427T^ were identified in South Asian individuals through genetic screens, with no detailed family studies (Banerjee et al., 2012; Bhattacharjee et al., 2007). In the gnomAD database, A427T is listed with a total allele frequency of 2.23×10^−5^, and is most prevalent in South Asian populations (allele frequency 1.43×10^−4^) (Table S1) (Chen et al., 2024).

### OLF^Q368X^ is purified in a misfolded state, whereas OLF^A427T^ adopts a near-native structure

Our laboratory developed a robust expression and purification protocol for producing many missense variants of the isolated OLF domain for biophysical and structural characterization (Burns et al., 2011; Scelsi et al., 2023). To date, only missense variants associated with the most severe disease phenotype, e.g. OLF^P370L^ and OLF^W286R^, could not be isolated in a soluble form for any *in vitro* measurements (Donegan et al., 2015). Similarly to these severe disease variants, OLF^Q368X^ produced only a misfolded aggregate characterized by elution from a size exclusion column in the void volume (Fig. S1A,B) with a high thioflavin T (ThT) fluorescence signal suggestive of amyloid (Fig. S1C). Thus, although we considered the possibility that an alternative stable tertiary structure might be adopted, perhaps one comprising the available blades of the propeller as a β-barrel, this was not the case.

Previously, we biophysically characterized OLF^A427T^ (Donegan et al., 2012; Hill et al., 2014). Purified OLF^A427T^ slowly forms amyloid-like ThT-positive aggregates at 36°C (Hill et al., 2014), a temperature at which OLF^WT^ does not aggregate. In addition, the midpoint of unfolding for OLF^A427T^ is 47.8°C, just above the previously identified 47°C cutoff for pathogenic OLF variants (Scelsi et al., 2023). In summary, both OLF^Q368X^ and OLF^A427T^ are biophysically distinct from OLF^WT^ and form ThT-positive aggregates.

To identify structural defects at the molecular level, we solved the crystal structure of OLF^A427T^ to 1.45 Å resolution (Fig. 1; Table S2). The structure confirms that the overall OLF five-bladed β-propeller, including the dinuclear metal cluster in the central hydrophilic cavity, remains intact. However, several deviations from OLF^WT^ are apparent. Typically, OLF^WT^ crystallizes with one polypeptide chain in the asymmetric subunit, in line with solution fractionation as a monomer (Huard et al., 2019). By contrast, OLF^A427T^ has four crystallographically independent copies of OLF^A427T^ [chains A-D, Fig. 1A; root mean squared deviation (r.m.s.d.)**=**0.21–0.23 Å], with two pairs of different deviations near the mutation site at residue 427, between chains A and B (blue, Fig. 1A,C,E; r.m.s.d.=0.23 Å) and chains C and D (pale cyan, Fig. 1A,D,E; r.m.s.d.=0.21 Å). Loop_440-446_ (green, Fig. 1B) could not be modeled in chains A and B, whereas in chains C and D the loop could be modeled and is shifted by ∼5 Å. Loop_469-475_ (yellow, Fig. 1B) could also not be modeled in chains A and B but retains WT-like configuration in chains C and D. Inspection of the site of mutation showed a new hydrogen bonding pattern due to the introduction of Thr427 that explains the new conformations of Loop_440-446_ (Fig. S2). Namely, in all four chains of the OLF^A427T^ structure, a water molecule forms a hydrogen bonding interaction with the γ-hydroxyl of the Thr side chain and appears to orient the backbone carbonyl of Ser440 into a distinct conformation compared to that of OLF^WT^ (Fig. S2B-E).

**Fig. 1.**
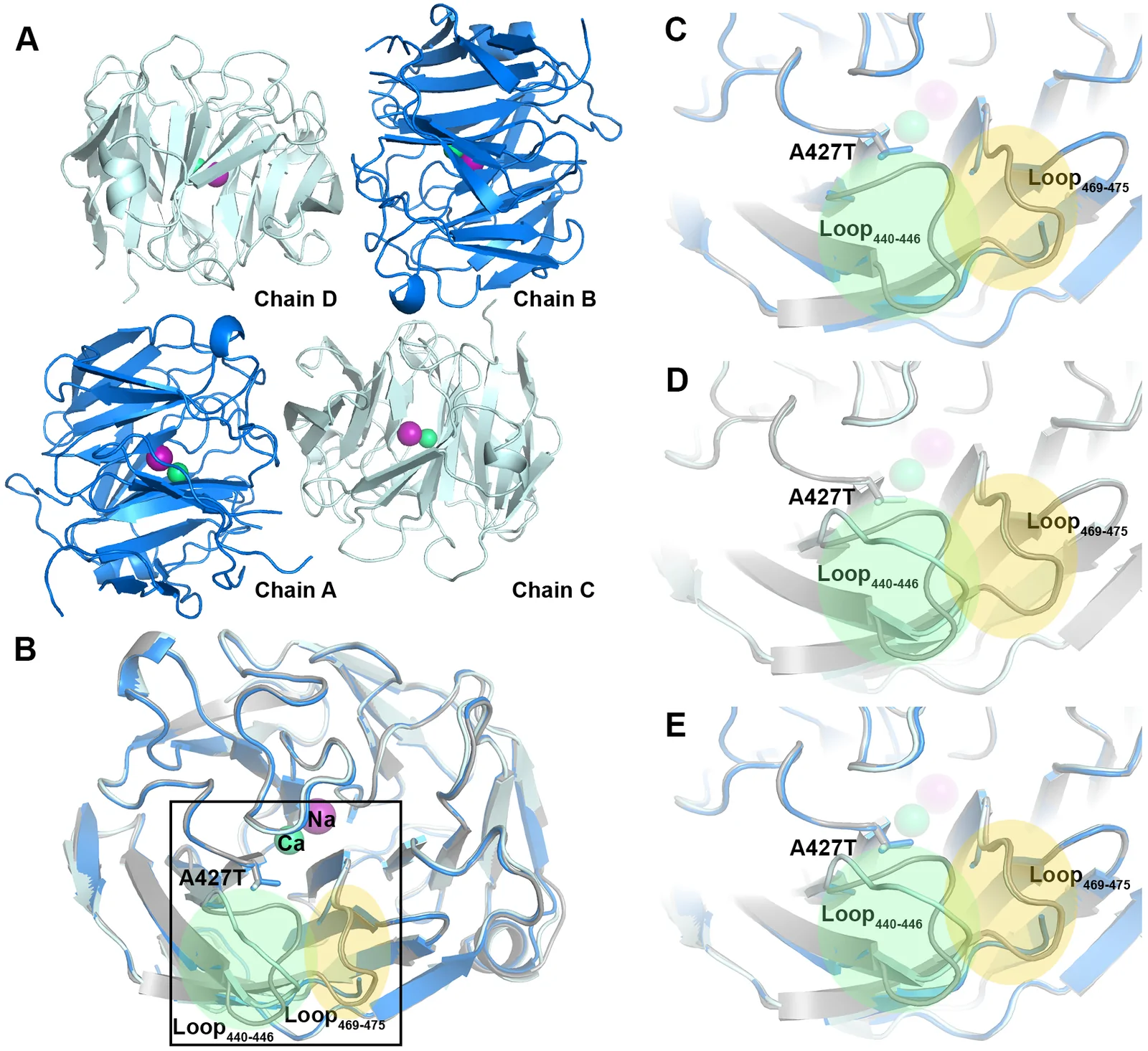
**Crystal structure of olfactomedin (OLF)^A427T^ shows differences in loop configurations.** (A) Arrangement of the protein in the crystal, with four monomers in the asymmetric unit. (B) Superposition of OLF^WT^ (PDB ID 8FRR, gray), OLFA^427T^ chain A (blue) and OLFA^427T^ chain C (pale cyan), with loops of interest [Loop_440-446_ (green) and Loop_469-475_ (yellow)] highlighted. (C) Zoom-in on superposition of OLF^WT^ and OLFA^427T^ chain A, showing unmodeled Loop_440-446_ and Loop_469-475_. (D) Zoom-in on superposition of OLF^WT^ and OLFA^427T^ chain C, showing structured Loop_440-446_ and Loop_469-475_. (E) Zoom-in on superposition of OLF^WT^, OLFA^427T^ chain A and OLFA^427T^ chain C. The Ala427Thr mutation pushes Loop_440-446_ up and out compared to OLF^WT^. Loop_469-475_ is unmodeled in chains A and B but retains a WT-like conformation in chains C and D. One crystal containing trillions of molecules was used for the experiment. See also crystallographic data and refinement statistics in Table S2.

The loss of electron density in loops of chains A and B near Thr427 is indicative of increased dynamics compared to that in OLF^WT^. Thermal B-factors are increased in OLF^A427T^ compared to those in OLF^WT^ at Loop_440-446_ and Loop_469-475_ (Fig. S3A-E). In addition, in chains A and B of OLF^A427T^, residues near the side helix (Gly292-Asp294 and Gln309-Tyr311), more than 20 Å from Thr427, have increased thermal B-factors. The side helix of OLF has been observed to shift and become more dynamic in response to other remote amino acid substitutions (Patterson-Orazem et al., 2019) and may suggest a common mechanism of long-range accommodation for residue changes in OLF. Overall, although structural perturbations and increased dynamics are apparent in OLF^A427T^, they do not appear severe as seen in disease mutants OLF^D380A^ and OLF^I499F^ (Saccuzzo et al., 2024), which are intractable by crystallography.

### Myocilin^WT^ is secreted, whereas disease variant myocilin^Y437H^ is retained intracellularly, in TM-1 cells

Cellular secretion assays evaluate the likelihood of pathogenicity of a given myocilin variant as a function of secretion – namely, by comparing the extent of myocilin in spent media and the relative abundance of intracellular detergent-insoluble (i.e. aggregated) and detergent-soluble (i.e. not aggregated) species, evaluated using immunoblotting (Zhou and Vollrath, 1999). In previous studies, we and others have shown that pathogenic myocilin mutants such as P370L, predominantly localize to the detergent-insoluble fraction (Liu and Vollrath, 2004; Scelsi et al., 2023; Stothert et al., 2014, 2017). Less severe variants of myocilin secrete to a lower extent than myocilin^WT^ but may accumulate more in the soluble than the insoluble fraction (Scelsi et al., 2023).

To avoid potential pitfalls due to robust overexpression and different proteomes in model cell lines, here, we used TM-1 cells that stably express doxycycline (dox)-inducible constructs of full-length myocilin^WT^, myocilin^Y437H^, myocilin^Q368X^ or myocilin^A427T^ to evaluate variant myocilin behavior. These cells were constructed as previously reported (Itakura et al., 2015) but were generated anew (see Materials and Methods). Consistent with early reports (Itakura et al., 2015), our western blots showed robust secretion of myocilin^WT^ and impaired secretion of the disease variant myocilin^Y437H^ (Fig. 2A; Fig. S4A). Further processing of our lysates into detergent-soluble and detergent-insoluble fractions revealed low levels of myocilin^WT^ and undetectable levels of myocilin^WT^, respectively. By contrast, myocilin^Y437H^ was present at low levels in both fractions (Fig. 2A; Fig. S4B,C). We note that although we treated the TM-1 cells with increasing levels of dox (1, 2 and 5 μg/ml), these observations did not appear to be dose dependent (Fig. S4D,E). Consistent with immunoblotting, immunofluorescence staining showed that myocilin^Y437H^ was present at relatively higher levels intracellularly than myocilin^WT^, but these differences did not reach statistical significance (Fig. 2B; Fig. S4H). Finally, cycloheximide chase experiments monitoring total myocilin levels showed that the half-life of insoluble myocilin^Y437H^ was ∼6 h, compared to <30 min for myocilin^WT^ (Fig. 2C,D; Fig. S5A,B). Corresponding immunofluorescence imaging confirmed an overall decrease in intracellular myocilin^WT^ as well as reduced colocalization in the ER at the conclusion of the 6-h chase period (Fig. S5C,D). Interestingly, even though the intensity of aggregate staining decreased over the 6-h chase period (Fig. S5C,E), we observed a statistically significant increase in colocalization of myocilin^WT^ with the ER marker calnexin and aggregate stain Proteostat (Fig. S5C,F,G), suggesting that any remaining intracellular myocilin^WT^ is misfolded and on path to be degraded. By contrast, and consistent with its long half-life, there were no changes in intensity or colocalization between the start and end points of the cycloheximide chase experiment for myocilin^Y437H^ (Fig. S5C-G).

**Fig. 2.**
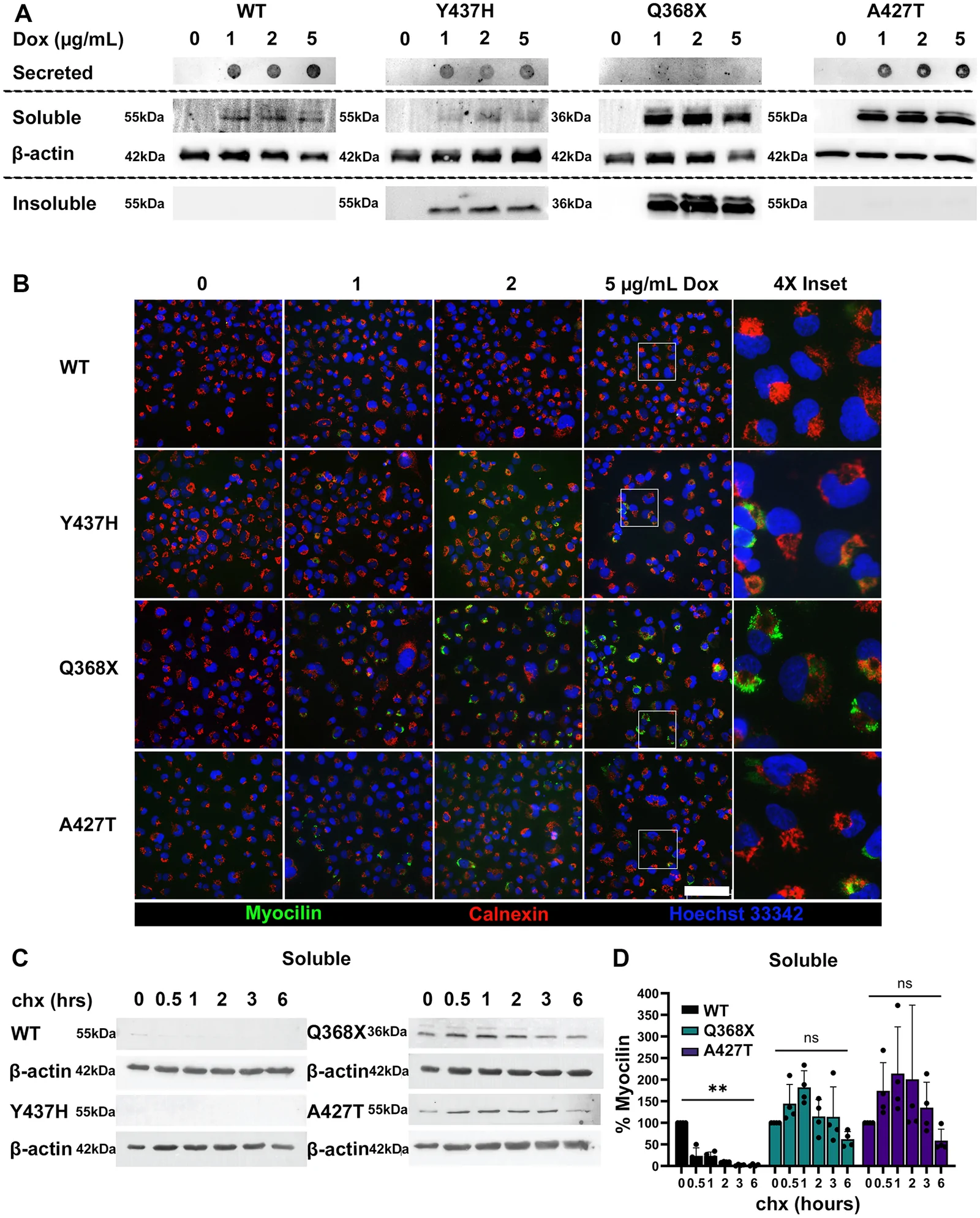
**Secretion, intracellular retention and degradation of myocilin variants in doxycycline-inducible trabecular meshwork cells*.*** (A) Expression of myocilin^WT^, myocilin^Y437H^, myocilin^Q368X^ or myocilin^A427T^ was induced with increasing doses of doxycycline (dox; 1, 2 and 5 μg/ml) (*n*=3). Myocilin secretion was assayed by dot blot analysis, while intracellular retention and aggregation was detected by western blot analysis of detergent-soluble and insoluble intracellular fractions. (B) Myocilin intracellular location was ascertained by immunocytochemistry, after inducing with 0, 1, 2 and 5 μg/ml dox, for myocilin (green) (*n*=3-4) and endoplasmic reticulum (ER) marker calnexin (red) (*n*=2-3). Scale bar: 100 μm. 4× zoomed views of 5 μg/ml dox-treated cells (white boxes) are shown in the far-right column. (C) Immunoblotting of detergent-soluble fractions (*n*=3-4) induced with 2 μg/ml dox and treated with cycloheximide (chx) for 6 h. β-actin served as a load control. (D) Quantification of C, with percentage myocilin shown relative to *t*=0 h in chx chase experiments. Because there was no detectable soluble Y437H, comparison to A427T/Q368X was not conducted. Error bars represent s.d. Data were analyzed by two-way ANOVA (ns, not significant; **P≤0.01).

### In TM-1 cells, myocilin^Q368X^ exhibits a severe misfolding phenotype, whereas myocilin^A427T^ exhibits a mild misfolding phenotype

Like myocilin^Y437H^, myocilin^Q368X^ demonstrated impaired secretion relative to myocilin^WT^ (Fig. 2A; Fig. S4A). As seen in western blots, myocilin^Q368X^ was present in both detergent-soluble and -insoluble fractions, at higher levels than for myocilin^Y437H^ (Fig. 2A; Fig. S4B,C). As above, the myocilin^Q368X^ results were dose independent (Fig. S4F) and agreed with the initial characterization of this cell line (Itakura et al., 2015). Immunofluorescence imaging (Fig. 2B; Fig. S4H) supported the high levels of intracellular myocilin^Q368X^ seen in western blots. However, colocalization of calnexin and myocilin^Q368X^ was not statistically significant (Fig. 2B; Fig. S4I), suggesting that intracellular myocilin^Q368X^ was not accumulating in the ER. Visual inspection revealed that intracellular myocilin^Q368X^ has a more punctate appearance than myocilin^Y437H^ (Fig. 2B). These puncta may be cytosolic aggresomes formed as a result of the inability to efficiently degrade myocilin^Q368X^. This would agree with the high levels of insoluble (i.e. aggregated) myocilin^Q368X^ seen in Fig. 2A. Overall, myocilin^Q368X^ is retained intracellularly like myocilin^Y437H^, but, unlike myocilin^Y437H^, appears to fractionate as soluble and insoluble species and appears morphologically distinct in immunofluorescence.

Unlike myocilin^Y437H^ or myocilin^Q368X^, myocilin^A427T^ was secreted, although to an extent lower than myocilin^WT^ (Fig. 2A; Fig. S4A). In the detergent-soluble fraction, myocilin^A427T^ was present at a level similar to that for myocilin^Q368X^ (Fig. 2A, Fig. S4B). These results are in line with previous studies of myocilin^A427T^, in which overexpression in TM-1 (Filla et al., 2002; Itakura et al., 2015) or COS-7 (Gobeil et al., 2006) cells showed myocilin^A427T^ to be present in secreted and soluble fractions. Like myocilin^WT^, myocilin^A427T^ was not readily detected in the detergent-insoluble fraction (Fig. 2A; Fig. S4C), and, like all other variants tested in TM-1 cells, these changes were dose independent (Fig. S4G). Similarly, immunofluorescence imaging (Fig. 2B; Fig. S4H) showed that myocilin^A427T^ did not appear to remain in the ER as its colocalization with calnexin was lower (*P*<0.05) than that of myocilin^WT^ (Fig. S4I).

Cycloheximide chase experiments revealed a longer lifetime of intracellular soluble myocilin of both myocilin^A427T^ and myocilin^Q368X^ than either myocilin^WT^ or myocilin^Y437H^ (Fig. 2C,D). Soluble myocilin^Q368X^ and myocilin^A427T^ was still present after 6 h (Fig. 2C,D). Further analysis of the insoluble fraction showed that only detergent-insoluble myocilin^A427T^ was degraded over the 6-h experiment (Fig. S5A,B). Immunostaining showed no statistically significant degradation of any variant over the 6 h measured in colocalization or aggregation (Fig. S5C-G). Morphological changes observed in nuclei of myocilin^Q368X^-expressing cells treated with cycloheximide, which was seen across all views, may indicate cell death or necrosis (Atale et al., 2014). In summary, cycloheximide chase data support results from immunoblotting and immunofluorescence imaging and confirm that the misfolded aggregate is challenging to degrade.

### Myocilin^WT^, but not disease variant myocilin^Y437H^, can be rerouted from ERAD to autophagic clearance

The lack of clearance of disease-causing myocilin variants is due to impaired ERAD (Huard et al., 2019; Suntharalingam et al., 2012). To further clarify the differential degradation pathways used by myocilin variants, we treated the TM-1 cells stably expressing myocilin^WT^ or variants with a proteasomal inhibitor (MG132) or an autophagy inhibitor [bafilomycin A1 (BafA1)]. The cellular secretion assay was employed again to evaluate any post-treatment change in myocilin abundance in media, detergent-soluble and detergent-insoluble fractions, by immunoblotting. Then, immunocytochemistry was used to deduce intracellular localization, e.g. with calnexin to determine ER retention or ATG5 to determine recruitment to phagophores (Walczak and Martens, 2013).

When TM-1 cells expressing modest levels of myocilin^WT^ were treated with MG132, myocilin^WT^ did not accumulate intracellularly. In immunoblot analyses, compared to DMSO vehicle control, MG132 treatment decreased secreted (*P*<0.001) and intracellular soluble myocilin^WT^, but did not change the levels of detergent-insoluble myocilin^WT^ (Fig. 3A; Fig. S6A). Similarly, in immunofluorescence imaging, myocilin^WT^ was found at relatively low levels intracellularly and colocalized with calnexin (Fig. 3B; Fig. S6B,D,E). Upon treatment with MG132, there was no change in calnexin colocalization, but there was a statistically significant increase (*P*<0.05) in colocalization with Proteostat and ATG5, indicating that, upon MG132 treatment, myocilin^WT^ aggregates are routed for degradation by the autophagy–lysosome pathway (Walczak and Martens, 2013) (Fig. 3C; Fig. S6C,D,F).

**Fig. 3.**
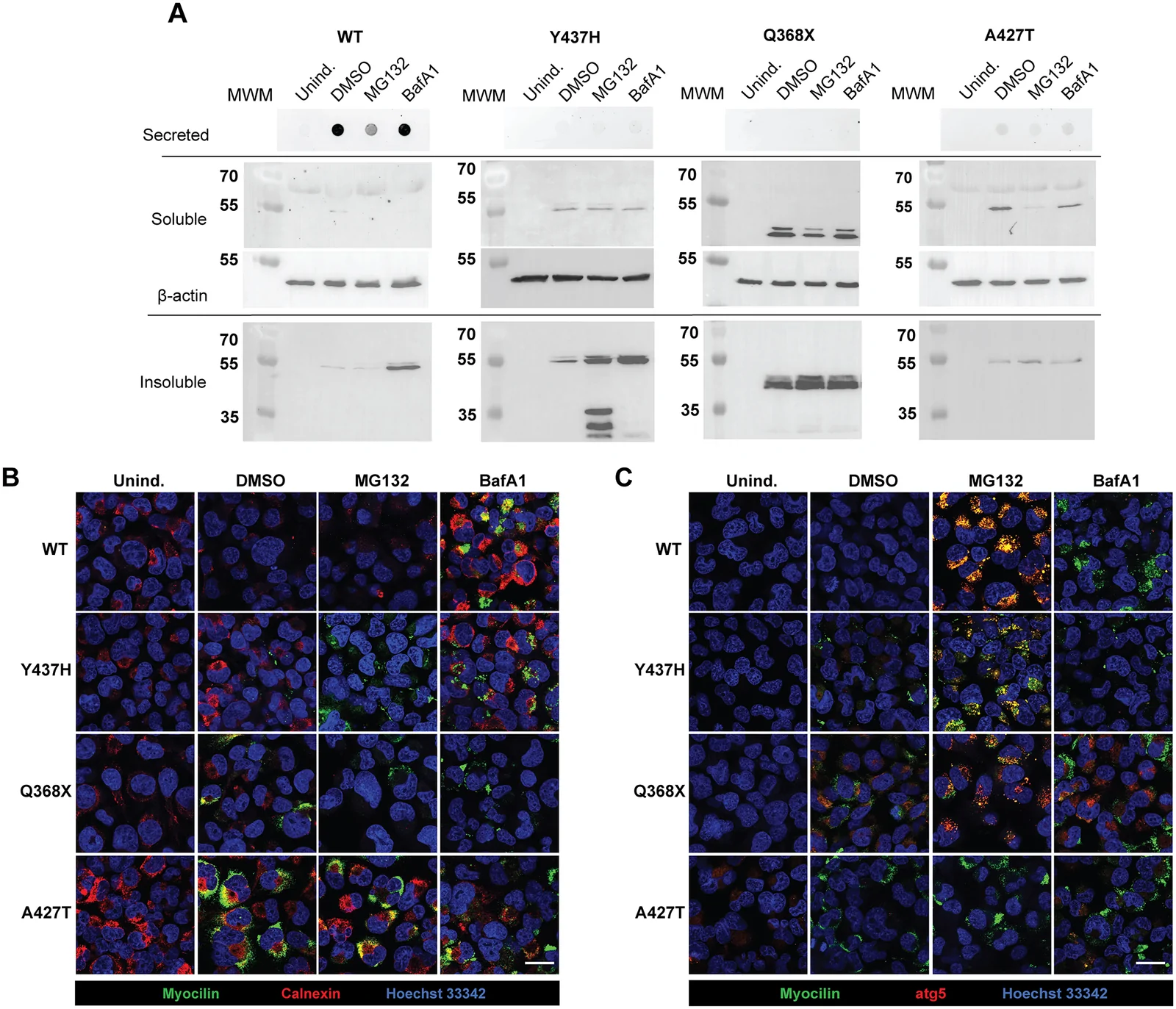
**Cellular trafficking of myocilin variants in doxycycline-inducible trabecular meshwork cells*.*** (A) Immunoblotting of secreted, detergent-soluble and detergent-insoluble myocilin^WT^, myocilin^Y437H^, myocilin^Q368X^ or myocilin^A427T^ induced with 2 μg/ml dox and treated with MG132 and bafilomycin A1 (BafA1) (*n*=3-4) with uninduced and vehicle (DMSO) controls. Secreted myocilin was assayed by dot blot analysis, while intracellular fractions were assessed by western blot analysis. β-actin served as a load control for the detergent-soluble fraction. (B) Immunocytochemistry of myocilin^WT^ and variants induced with 2 μg/ml dox and treated with MG132 and BafA1 with uninduced and vehicle (DMSO) controls (*n*=3, 2). Myocilin (green), calnexin (red) and nuclei (blue). Scale bar: 20 μm. (C) Immunocytochemistry of myocilin^WT^ and variants induced with 2 μg/ml dox and treated with MG132 or BafA1 with uninduced and vehicle (DMSO) controls (*n*=3 experimental groups, *n*=2 control groups). Myocilin (green), ATG5 (red) and nuclei (blue). Scale bar: 20 μm. See Fig. S6 for Proteostat staining of the same samples.

Conversely, treatment with BafA1 showed that proteasomal clearance alone was insufficient to handle myocilin^WT^ degradation. In immunoblot analyses, treatment with BafA1 led to no change in secreted or soluble myocilin^WT^ but an increase in insoluble myocilin^WT^ (Fig. 3A; Fig. S6A). Accordingly, in immunofluorescence imaging, there was a statistically significant increase (*P*<0.01) in fluorescence intensity corresponding to myocilin^WT^ compared to that with DMSO vehicle control (Fig. S6D,E), but no statistically significant change in colocalization with calnexin, ATG5 or Proteostat (Fig. 3B,C; Fig. S6B-D,F).

For myocilin^Y437H^, neither MG132 nor BafA1 treatment led to efficient protein degradation. The addition of DMSO as vehicle led to the detection of a soluble myocilin^Y437H^ species compared to no DMSO, but this species was unchanged with either MG132 or BafA1 treatment. The levels of the detergent-insoluble fraction were higher for both treatments, but this was statistically significant (*P*<0.05) only for BafA1 treatment (Fig. 3A; Fig. S6A). However, we note that the detergent-insoluble species included truncated species for the MG132 treatment (Fig. 3A; Fig. S6A) not included in the calculation. Immunofluorescence levels of intracellular myocilin^Y437H^ were elevated for both treatments, but these did not reach statistical significance (Fig. S6D,E). Because the myocilin^Y437H^ levels did not statistically increase (Fig. S6D,E), but the Proteostat signal increased with MG132 treatment (*P*<0.05; Fig. S6F), we infer that the Proteostat-positive aggregate is recalcitrant to degradation. This finding is in line with prior findings that myocilin^Y437H^ forms amyloid-like aggregates (Ma et al., 2025; Scelsi et al., 2023). In addition, treatment with either MG132 or BafA1 yielded no change in the extent of colocalization between calnexin and myocilin^Y437H^ (Fig. 3B; Fig. S6B). However, with MG132 treatment, there was a statistically significant increase (*P*<0.05) in colocalization with ATG5 (Fig. 3C; Fig. S6C). This result suggests that impairment of proteasomal degradation with MG132 leads TM-1 cells to attempt to engage autophagy, similar to the scenario with myocilin^WT^, although the attempt was unsuccessful in this case, as confirmed by an increase in insoluble myocilin^Y437H^. Overall, the defect in myocilin^Y437H^ degradation is reminiscent of other amyloid disease-relevant proteins such as α-synuclein, which is related to Parkinson's disease (Cuervo et al., 2004).

### Neither myocilin^Q368X^ nor myocilin^A427T^ can be efficiently rerouted from ERAD to autophagic clearance

As seen in immunoblot analyses, treatment with MG132 caused visible, although not statistically significant, increases in insoluble myocilin^Q368X^ and myocilin^A427T^, suggesting that, like for myocilin^Y437H^, autophagic clearance cannot efficiently compensate for impaired proteasomal degradation. In immunocytochemistry, myocilin levels and colocalization with calnexin or ATG5 were unchanged for either treatment (Fig. 3B,C; Fig. S6B,C,E). However, Proteostat colocalization was significantly increased (*P*<0.001) when the proteosome was inhibited (Fig. S6D,F), which agrees with the increase in insoluble myocilin^Q368X^ observed in immunoblotting. In specific support of impaired ERAD, inhibiting Grp94 in myocilin^A427T^-expressing TM-1 cells elicited dose-dependent degradation of myocilin^A427T^ (Fig. S7), similar to what was observed previously for other disease variants in cells (Huard et al., 2018; Stothert et al., 2014; Suntharalingam et al., 2012) and in mice (Stothert et al., 2017).

### Transcriptomics analysis bolsters similarity in TM-1 cellular response upon mutant myocilin expression

Lastly, we evaluated transcriptional effects of the variants in TM-1 cells expressing the four myocilins. Compared to cells without induction, in cells expressing myocilin^WT^, the lysosomal degradation pathway is upregulated, corroborating our result that TM-1 cells can shuttle myocilin^WT^ to autophagy under stress (Fig. 4; Table S3). In addition, in line with its secretion profile, pathways for N-glycan synthesis and protein processing in the ER are also enriched in the dataset for myocilin^WT^ (Fig. 4; Table S3A). Comparing myocilin^Y437H^ to myocilin^WT^, some of the genes in the ubiquitin-mediated proteolysis pathway are downregulated while others are upregulated, whereas genes in lysosome and protein processing in the ER pathways are all downregulated (Fig. 4; Table S3B). For myocilin^Q368X^, ubiquitin-mediated proteolysis is again upregulated compared to myocilin^WT^, and the downregulated pathways are the same as for myocilin^Y437H^ (Fig. 4; Table S3D). Finally, comparison of myocilin^A427T^ to myocilin^WT^ revealed upregulated pathways related to ubiquitin-mediated proteolysis as well as the proteasome, whereas those of the lysosome and protein processing in the ER are downregulated (Fig. 4; Table S3C). In summary, transcriptomics recapitulates our overall conclusion that myocilin^Q368X^ and myocilin^A427T^ exhibit similarities with disease variant myocilin^Y437H^, in that they alter pathways that result in an inability to engage autophagic clearance in TM-1 cells.

**Fig. 4.**
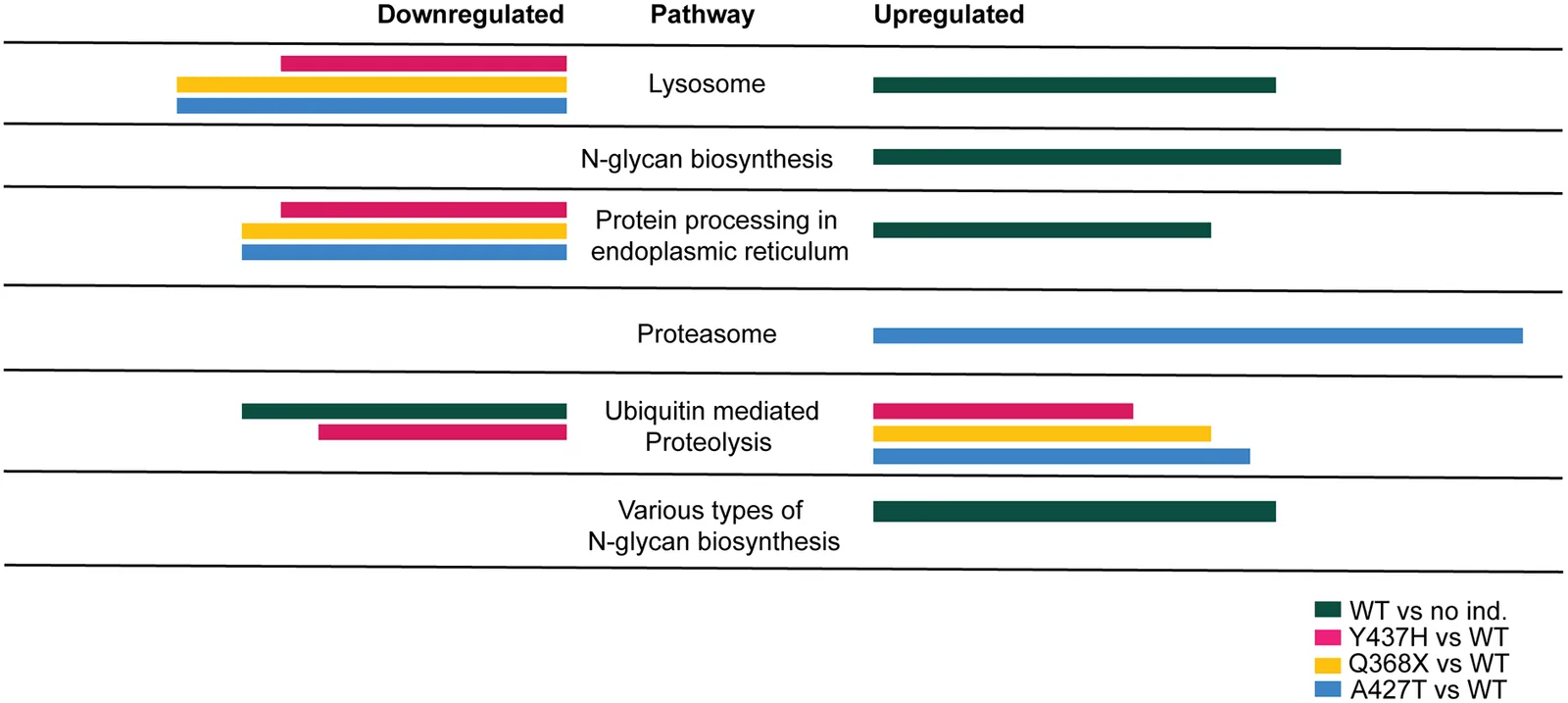
**Overall effect of myocilin expression on key pathways.** Visualization based on data presented in Table S3, scaled for fold enhancement. Transcriptomics was conducted for each mutant in independent inductions (*n*=3) and compared to cells not induced (*n*=3).

## DISCUSSION

Our study shows that myocilin misfolding occurs on a continuum, and impaired myocilin degradation is not limited to thermally unstable missense variants. Upon proteasome inhibition, only myocilin^WT^ does not accumulate, whereas, when autophagy is inhibited, myocilin^WT^ and myocilin^Y437H^ accumulate, but there is no change in the degradation of myocilin^Q368X^ or myocilin^A427T^. Myocilin^WT^ and myocilin^Y437H^ colocalize with ATG5, suggesting that TM-1 cells unsuccessfully attempt to reroute myocilin^Y437H^ from ERAD to autophagy. However, neither myocilin^Q368X^ nor myocilin^A427T^ colocalizes with ATG5, suggesting that TM-1 cells rely on the proteasome for these variants.

Myocilin^Y437H^, myocilin^A427T^ and myocilin^Q368X^ variants are vastly different proteins, but they all accumulate Proteostat- and ThT-positive intracellular species in cells and *in vitro*, respectively. Surprisingly, myocilin^A427T^ posed a challenge to TM-1 cells akin to disease variants. Myocilin^A427T^ harbors a conservative OLF mutation that introduces a minor decrease in thermal stability and structural perturbations compared to OLF^WT^. In addition, because OLF^A427T^ crystallized, one can assume that a largely homogeneous population of protein states is present in solution (McPherson and Gavira, 2014). Thus, we infer that even minimal defects in myocilin^A427T^ are sufficient to be recognized as aberrant by the ER chaperone system (Flagg et al., 2023) and prevent myocilin^A427T^ from being secreted at WT levels. The accumulation of myocilin^A427T^ then increases its local intracellular concentration, which may allow for misfolding events to nucleate and propagate more readily than when the concentration is lower, as would be the case for myocilin^WT^. The similarity of myocilin^A427T^ to disease mutants is strengthened by the finding that treatment with a Grp94 inhibitor allows for degradation, presumably via autophagy, as we found previously for disease mutants (Huard et al., 2018; Stothert et al., 2014; Suntharalingam et al., 2012). On the other end of the spectrum, OLF^Q368X^, similarly to studied missense disease variants, cannot be expressed and purified in a soluble state for crystallization. However, myocilin^Q368X^ differs from missense variants in that only half the domain is translated, so the aggregate formed necessarily includes fewer residues and would presumably be recognized by different molecular chaperones than missense variants (Gidalevitz et al., 2013). As rerouting myocilin^WT^ to autophagic clearance is possible when proteasomal degradation is inhibited, future studies will focus on elucidating the specific rerouting mechanisms accessible to mutant myocilin, such as ER-to-lysosome-associated degradation (ERLAD), macro-ER-phagy, micro-ER-phagy and LC3-mediated vesicular transport pathways (Molinari, 2021).

Given that myocilin^A427T^ and myocilin^Q368X^ are neither associated with early onset glaucoma nor consistently appear in patients with glaucoma, our findings further suggest that the extent of pathogenicity may be influenced by a secondary factor, such as background genetics or by environmental factors. Indeed, genetic factors are plausible as myocilin^Q368X^ has been found to be co-inherited with other genes that confer higher risk for glaucoma (O'Gorman et al., 2019; Wiggs et al., 2013; Willoughby et al., 2004; Young et al., 2012) (Table S1). Furthermore, environmental factors can make more common and low phenotypic effect variants hard to pinpoint (Manolio et al., 2009). For example, use of glucocorticoids can induce expression of myocilin in TM cells (Nguyen et al., 1998), and steroids can lead to ocular hypertension (Fini et al., 2017; Overby and Clark, 2015), although these effects are not thought to be directly correlated (Patel et al., 2017). Other environmental factors relevant to glaucoma and TM dysfunction that could make cells more susceptible to protein misfolding include increased reactive oxygen species and mechanical stress (Itakura et al., 2015; Yan et al., 2024), or aging (Yu et al., 2025). Individualized vulnerabilities could push mild variants such as myocilin^A427T^, and potentially even myocilin^WT^, beyond their proteostatic threshold.

In summary, contributions of myocilin to glaucoma pathogenicity may be more expansive than previously appreciated. There are likely to be other such threshold variants of myocilin that behave similarly to myocilin^A427T^ and premature stop variants with behavior like myocilin^Q368X^. Our study highlights the need for genetic screening, as well as an exploration of environmental or lifestyle factors (Almarzouki, 2024; Elhusseiny and Aref, 2025) and other aspects of genetics (Gao et al., 2022) that may increase susceptibility to developing glaucoma. More broadly, our study supports autophagy manipulation as a therapeutic strategy to treat proteostasis decline in TM cells, which is thought to contribute to the causal risk factor for glaucoma – namely, IOP elevation (Shim and Liton, 2022).

## MATERIALS AND METHODS

### OLF expression and purification

OLF^Q368X^ and OLF^A427T^ were recombinantly expressed and purified as described previously (Donegan et al., 2015) with minor modifications. Briefly, Rosetta-gami 2(DE3) cells (Novagen) transformed with plasmids were grown in Superior broth (US Biological) supplemented with 60 μg/ml ampicillin and 34 μg/ml chloramphenicol. Cultures were induced at an optical density at 600 nm (OD_600_) of 1.5 at 18°C with 0.5 mM isopropyl-β-D-thiogalactopyranoside and 100 mM CaCl_2_, and cells were allowed to grow for 16 h. The cells were pelleted, flash frozen in liquid nitrogen and stored at −80°C. Protein was purified by amylose affinity chromatography and size-exclusion chromatography, as reported previously (Donegan et al., 2012). For OLF^Q368X^, the void volume peak of the Superdex75 column was pooled as the MBP-OLF^Q368X^ aggregate and stored at 4°C in PGF buffer (10 mM Na_2_HPO_4_/KH_2_PO_4_, 0.2 M NaCl, pH 6.8). MBP-OLF^Q368X^ aggregates were concentrated using a 30 kDa MWCO Amicon filter to a concentration of ∼40 μM. Concentration was determined by Beer's law using A280 and a predicted extinction coefficient of 99,240 M^−1^cm^−1^. For OLF^A427T^, following an amylose affinity and size exclusion column, MBP was cleaved from OLF using TEV protease overnight at room temperature, and OLF^A427T^ was isolated by size exclusion chromatography. Purified OLF^A427T^ was stored in PGF buffer (10 mM Na_2_HPO_4_/KH_2_PO_4_, 0.2 M NaCl, pH 6.8). Purity of proteins was assessed by standard 12% SDS-PAGE analysis using stain-free and Coomassie Blue-stained gels visualized with a Bio-Rad ChemiDoc MP Imaging System.

### Aggregation assays

Aggregation assays were conducted as described previously (Hill et al., 2014; Scelsi et al., 2025). In summary, 50 µl aliquots were prepared with 10 μM ThT and 30 μM protein. Samples of OLF^WT^ monomer, MBP, MBP-OLF^WT^ aggregates and MBP-OLF^Q368X^ aggregates were analyzed. ThT fluorescence measurements were acquired on a Horiba spectrofluorometer with an excitation wavelength of 440±2 nm and an emission wavelength of 485±2 nm. Samples were measured as analytical duplicates, and the average blank subtracted fluorescence value was recorded. Data are representative of two replicates.

### Crystallization and structure determination

Purified OLF^A427T^ (in 10 mM HEPES, pH 7.2 with 20 mM NaCl) was concentrated to 10 mg/ml. Crystals were grown by the hanging-drop method by equilibration against a reservoir containing 10% PEG 8000 (Hampton Research) and 0.05 M MgCl_2_. Crystals were cryo-cooled in a solution containing reservoir solution supplemented with 30% glycerol. X-ray diffraction datasets were collected on the NYX beamline 19-D at Brookhaven National Laboratory National Synchrotron Light Source II (NSLS-II) and were processed using HKL-2000 (Otwinowski and Minor, 1997). Structures were solved by molecular replacement using Phaser (McCoy, 2007), using OLF^WT^ (PDB ID 4WXQ) (Donegan et al., 2015) as the search model. The models were iteratively refined using Coot (Emsley et al., 2010) and Phenix.refine (Afonine et al., 2012). Figures were prepared in PyMOL. Data collection statistics are reported in Table S2.

### Lentiviral construction for myocilin transduction in human trabecular meshwork cells

Site-directed mutagenesis to create the human *MYOC^Y437H^*, *MYOC^Q368X^* and *MYOC^A427T^* mutations, and insertion of WT and mutant cDNAs into the lentiviral vector pSLIK-hygro (Invitrogen, Carlsbad, CA, USA) was previously described (Itakura et al., 2015). For packaging, lentiviral expression plasmids were co-transfected into HEK293T cells along with helper plasmids pMD.G1 and pCMVR8 (Shin et al., 2006). Culture medium containing viral particles was harvested after 48-72 h and stored at −70°C.

### Generation of stably transduced human trabecular meshwork cells

Immortalized trabecular meshwork (TM-1) cells were established from human TM cells isolated from a 30-year-old donor eye as previously described using SV40 origin defective vector (Faralli et al., 2019; Filla et al., 2002; Murnane et al., 1985; Polansky et al., 1979, 1984). Cells were validated using short tandem repeat (STR) analysis (Masters et al., 2001). Viral particles described above were used to infect immortalized TM-1 cells. Cell cultures were infected at a low multiplicity of infection (MOI; 50) to ensure <30% infection frequency. Cells stably transduced with lentiviral expression vectors were selected by treating the cultures with 150 μg/ml hygromycin (Invitrogen) for 3 weeks. For induction of myocilin, TM-1 cells of stably transduced lines were seeded in six-well plates at 200,000 cells per well. To induce the transgene, cells were then treated with dox (1-5 μg/ml) for up to 7 days.

### Cell culture

Cells were maintained in low-glucose (1 g/l) Dulbecco's modified Eagle medium (DMEM) with glutamine (Gibco) supplemented with 10% fetal bovine serum (FBS; Hyclone) and 1-2% penicillin–streptomycin (Gibco) at 37°C per consensus recommendations (Keller et al., 2018). Medium was supplemented with 200 μg/ml hygromycin (Corning) to keep cells in selection. Cells were not otherwise authenticated or tested for contamination.

### Secretion assay

TM-1 cells stably transduced with myocilin (50 MOI) were seeded in six-well plates at equivalent cell counts/replicate. The next day, the cells were treated with dox (Thermo Fisher Scientific) in serum-free media to induce myocilin expression. For dose-dependency experiments, cells were treated with 0, 1, 2 and 5 μg/ml dox for 48 h. For drug treatment experiments, cells were treated only with 2 μg/ml dox. After 48 h, medium was removed, and cells were treated with inhibitors in media at desired concentrations for 16 h or otherwise specified. Inhibitors included MG132 at 10 μM (Sigma-Aldrich M7499), BafA1 at 100 nM (Sigma-Aldrich SML1661-.1), cycloheximide at 100 μg/ml (Sigma-Aldrich 239765) and 4-Br-BnIm (kind gift from the Blagg Laboratory, University of Notre Dame, Notre Dame, IN, USA) at 3, 30 and 200 μM. After treatment duration, spent medium containing secreted myocilin was collected and treated with protease inhibitor cocktail containing one tablet of cOmplete EDTA-free protease inhibitor (Roche) and 1% (v/v) of phosphatase inhibitor cocktails II and III (Sigma-Aldrich). Cocktail was added at 1:100 ratio of protease inhibitor cocktail to collected medium. After washing with PBS, cells were lysed with Triton X-100 lysis buffer (100 mM Tris-HCl pH 7.4, 3 mM EGTA, 5 mM MgCl_2_, 0.5% Triton-X 100) supplemented with phenylmethylsulfonyl fluoride to a final concentration of 0.6 mM and protease inhibitor cocktail as described above at 1:100 ratio. Cells were lysed by rocking at 4°C for >2 h. Detergent-soluble (i.e. supernatant) and detergent-insoluble (i.e. pellet) fractions were separated by centrifugation at 16,000 ***g*** for 10 min at 4°C. The detergent-insoluble fraction was PBS washed before being resuspended in 2× Laemmli's Buffer+10% 2-mercaptaethanol and sonicating with a rod sonicator (Qsonica Q125) for 5 min with 10-s on/off pulses at 50% amplitude. Protein concentration of the spent media and detergent-soluble fractions was determined by BCA assay (Pierce).

### Immunoblotting

In preparation for western blot analysis, detergent-soluble samples were prepared in equal protein concentration within each experiment in a 2× Laemmli buffer containing 5-10% (v/v) β-mercaptoethanol. Samples were heated at 95°C for 5 min and loaded on either 12% tris-glycine gels made in house or 4-15% mini-PROTEAN TGX precast protein gels (Bio-Rad) in equal protein quantity within each fraction and experiment. Insoluble samples were heated at 95°C for an additional 15-20 min and loaded on gels in equal volume per lane. For dot blot analysis, cell media were prepared in equal protein concentration within each experiment. For immunoblot analysis, gels were transferred to methanol-activated PVDF membranes using a Trans-blot Turbo transfer system (Bio-Rad). For dot blot analysis, cell media samples were deposited in equal protein quantities onto Amersham Protran nitrocellulose membranes (Sigma-Aldrich) soaked in PBS buffer (Gibco) using a Bio-Dot microfiltration apparatus (Bio-Rad). Downstream western and dot blot steps were performed as described above. Membranes were blocked with 5% milk in PBS+0.5% Tween, washed and incubated with 1:1000 dilution of primary antibodies (mouse anti-myocilin, R&D Systems MAB3446 and rabbit anti-beta actin, Cell Signaling Technology 4970), followed by an additional wash and incubation with 1:1000-1:3000 dilution of secondary antibodies (anti-mouse StarBright Blue 520 and anti-rabbit StarBright Blue 700, Bio-Rad). After a final wash, blots were visualized using a ChemiDoc MP Imaging System (Bio-Rad). For dose-dependency experiments, quantification of western blots for soluble protein were performed using ImageJ (Schneider et al., 2012) by measuring the mean gray value for both myocilin and β-actin. Band density measurements were background subtracted, and soluble data were normalized to β-actin. Fold change was calculated for myocilin variants relative to myocilin^WT^ per fraction and doxycycline concentration, and relative to 1 μg/ml per fraction and variant. Data were analyzed by two-way ANOVA using GraphPad Prism. For cellular trafficking, cycloheximide chase and Grp94 inhibitor experiments, quantification of protein bands on blots was performed using ImageLab (Bio-Rad). Measurements of band density were normalized to β-actin for detergent-soluble fractions within each experimental treatment. Statistical significance was determined with two-way ANOVA within GraphPad Prism. Only within the same variant were treatments and conditions compared. Colocalization was assessed by Pearson's correlation coefficient of overlapping pixels after background signal correction.

### RNA-sequencing analysis

TM-1 cells stably transduced with the four myocilin variants (50 MOI) were cultured in duplicate in 250 ml, 75 cm^2^ tissue culture flasks (Falcon). Upon reaching confluency, the cells were treated with 2 μg/ml dox (Thermo Fisher Scientific) in serum-free media to induce myocilin expression. Two flasks of cells transduced with WT *MYOC* were treated with dox-free and serum-free media to serve as uninduced controls. After 24 h, cells were detached using 0.25% trypsin, 2.21 mM EDTA, 1× [−] sodium bicarbonate (Corning) and pelleted by centrifugation at 300 ***g*** for 5 min. Upon resuspension into fresh medium, an aliquot of each cell sample was dyed with an equal volume of Trypan Blue dye 0.40% (Bio-Rad) and counted on a TC20 Automated Cell Counter (Bio-Rad). Cells were then diluted, and a volume of each sample was aliquoted to create samples of 450,000 cells, which were pelleted, washed with PBS pH 7.4 (Gibco) and then flash frozen. Harvested TM-1 cells were processed and analyzed for significantly differentially expressed genes by Admera Health. Further analysis of the data for affected Kyoto Encyclopedia of Genes and Genomes (KEGG) pathways (Kanehisa et al., 2025) was conducted in ShinyGO v0.82 (Ge et al., 2020) using a false discovery rate (FDR) cutoff of 0.05.

### Immunocytochemistry

For dose-dependency experiments, human TM-1 cells stably transduced with myocilin (50 MOI) were seeded on glass coverslips (12 mm diameter, Fisher Scientific) coated with 2% gelatin (Sigma-Aldrich) in 24-well plates. The next day, cells were treated with 0, 1, 2 and 5 μg/ml dox to induce myocilin expression. Four separate replicate inductions were performed for myocilin^WT^, myocilin^Q368X^ and myocilin^A427T^; three were performed for myocilin^Y437H^. After 48 h, cells were washed with 1× PBS (Gibco), fixed with 10% formalin (Fisher Healthcare), permeabilized with 0.03% Triton X-100 (VWR Health Sciences) and blocked in 5% milk, before incubating with primary antibodies for >12 h (1:1000 rabbit anti-myocilin, Abcam ab41552, RRID: AB_776605; 1:1000 mouse anti-calnexin, Invitrogen MA3-027, RRID: AB_2069043). Following a PBS wash, cells were incubated with 1:1000 secondary antibodies (goat anti-rabbit IgG, Alexa Fluor 488, Invitrogen A11034, RRID: AB_2576217; goat anti-mouse IgG, Cyanine5, Invitrogen A10524, RRID: AB_10562712) for 1 h. Following another PBS wash, cells were incubated with 1:1000 Hoechst 33342 (Enzo Life Sciences ENZ51035K100) for ∼30 min. Cells were washed before fixing coverslips with ProLong^TM^ Diamond Antifade Mountant (Invitrogen) and allowed to cure for at least 24 h. Negative controls for each condition were treated with only the secondary antibodies and not the primary antibodies to eliminate non-specific antibody detection. Two to three positively and one to three negatively stained coverslips were prepared for each condition, and three to four representative images were acquired per coverslip at 40× magnification using a Leica DMB6 fluorescent microscope (Leica Microsystems). Brightness and contrast of all images in the panel was equivalently adjusted with Leica Application Suite X software and Adobe Photoshop. Quantification was conducted using ImageJ (Schneider et al., 2012) by measuring the mean gray value for both myocilin and Hoescht 33342 fluorescence after autothreshholding with the Triangle autothreshholding method. Myocilin fluorescence values were normalized to Hoechst 33342 fluorescence values. Outlier data were excluded by the interquartile method before averaging normalized data per replicate and condition. Average values were background subtracted with the average value for 0 μg/ml control for each variant. Fold change was calculated for myocilin variants relative to myocilin^WT^ per doxycycline concentration. Normalized and averaged data were analyzed by two-way ANOVA using GraphPad Prism. Analysis of colocalization of myocilin and calnexin was conducted using the EzColocalization ImageJ plugin (Schneider et al., 2012), with Triangle autothreshholding to measure the Pearson's correlation coefficient. Data were analyzed by two-way ANOVA using GraphPad Prism. Cells were stained for calnexin three out of four times for myocilin^WT^, myocilin^Q368X^ and myocilin^A427T^ and two out of three times for myocilin^Y437H^. As a result, myocilin^Y437H^ was excluded from the colocalization analysis.

For cycloheximide chase and cellular trafficking experiments, human TM-1 cells stably transduced with myocilin (50 MOI) were seeded on glass coverslips (12 mm diameter, Fisher Scientific) coated with 1.5 μg/ml poly-L-lysine (ScienCell 0403) in 24 well plates. The next day, the cells were treated with 2 μg/ml doxycycline (*n*=3 independent experiments). After a 16-h drug treatment, cells were washed with 1× PBS (Gibco), fixed with 10% formalin (Fisher Healthcare), permeabilized with permeabilization buffer containing 0.05% Triton X-100 (VWR Health Sciences) and blocked in 2% bovine serum albumin overnight before incubating with primary antibodies for 4 h (1:200 mouse anti-myocilin, R&D Systems MAB3446, RRID: AB_2148649; 1:200 rabbit anti-calnexin, Thermo Fisher Scientific PA5-34754, RRID: AB_2552106). Cells were incubated with 1:1000 secondary antibodies (goat anti-rabbit IgG, Alexa Fluor 488, Invitrogen A11034; goat anti-mouse IgG, Cyanine5, Invitrogen A10524) for 1 h. Following another PBS wash, cells were incubated with 1:1000 Hoechst 33342 and 1:2000 Proteostat (Enzo Life Sciences ENZ51035K100) for ∼30 min. Cells were washed before fixing coverslips with ProLongTM Diamond Antifade Mountant (Invitrogen) and allowed to cure for at least 24 h. Coverslips were prepared for each condition including uninduced cells, with each condition having two coverslips per each of two biological replicates with three to four representative images acquired per coverslip. Images were collected at 63× with a Zeiss 700 Laser Scanning Microscope with the same laser power conditions across all samples in trafficking experiments and by cell line for cycloheximide chase. Image channel brightness and gamma were equivalently adjusted with the Zen Blue software. Intensity quantification was conducted measuring the mean gray value of adjusted images normalized to Hoechst signal and background subtracted. Colocalization analysis was conducted using the JACoP plugin (Bolte and Cordelières, 2006) in ImageJ (Schneider et al., 2012). Statistical analysis was performed by two-way ANOVA with GraphPad Prism.

## Supplementary Material

10.1242/dmm.052821_sup1 Supplementary information

## References

[DMM052821C1] Afonine, P. V., Grosse-Kunstleve, R. W., Echols, N., Headd, J. J., Moriarty, N. W., Mustyakimov, M., Terwilliger, T. C., Urzhumtsev, A., Zwart, P. H. and Adams, P. D. (2012). Towards automated crystallographic structure refinement with phenix.refine. *Acta Crystallogr. D Biol. Crystallogr.* 68, 352-367. 10.1107/S0907444912001308 PMC3322595 22505256

[DMM052821C2] Aldred, M. A., Baumber, L., Hill, A., Schwalbe, E. C., Goh, K., Karwatowski, W. and Trembath, R. C. (2004). Low prevalence of MYOC mutations in UK primary open-angle glaucoma patients limits the utility of genetic testing. *Hum. Genet.* 115, 428-431. 10.1007/s00439-004-1171-1 15338275

[DMM052821C3] Allingham, R. R., Wiggs, J. L., De La Paz, M. A., Vollrath, D., Tallett, D. A., Broomer, B., Jones, K. H., Del Bono, E. A., Kern, J., Patterson, K. et al. (1998). Gln368STOP myocilin mutation in families with late-onset primary open-angle glaucoma. *Invest. Ophthalmol. Vis. Sci.* 39, 2288-2295. 9804137

[DMM052821C4] Almarzouki, N. (2024). Impact of environmental factors on glaucoma progression: a systematic review. *Clin. Ophthalmol.* 18, 2705-2720. 10.2147/OPTH.S484855 PMC11451402 39372224

[DMM052821C5] Atale, N., Gupta, S., Yadav, U. C. and Rani, V. (2014). Cell-death assessment by fluorescent and nonfluorescent cytosolic and nuclear staining techniques. *J. Microsc.* 255, 7-19. 10.1111/jmi.12133 24831993

[DMM052821C6] Banerjee, D., Bhattacharjee, A., Ponda, A., Sen, A. and Ray, K. (2012). Comprehensive analysis of myocilin variants in east Indian POAG patients. *Mol. Vis.* 18, 1548-1557. PMC3380904 22736945

[DMM052821C7] Bershtein, S., Mu, W., Serohijos, A. W., Zhou, J. and Shakhnovich, E. I. (2013). Protein quality control acts on folding intermediates to shape the effects of mutations on organismal fitness. *Mol. Cell* 49, 133-144. 10.1016/j.molcel.2012.11.004 PMC3545112 23219534

[DMM052821C8] Bhattacharjee, A., Acharya, M., Mukhopadhyay, A., Mookherjee, S., Banerjee, D., Bandopadhyay, A. K., Thakur, S. K., Sen, A. and Ray, K. (2007). Myocilin variants in Indian patients with open-angle glaucoma. *Arch. Ophthalmol.* 125, 823-829. 10.1001/archopht.125.6.823 17562996

[DMM052821C9] Bolte, S. and Cordelières, F. P. (2006). A guided tour into subcellular colocalization analysis in light microscopy. *J. Microsc.* 224, 213-232. 10.1111/j.1365-2818.2006.01706.x 17210054

[DMM052821C10] Burns, J. N., Orwig, S. D., Harris, J. L., Watkins, J. D., Vollrath, D. and Lieberman, R. L. (2010). Rescue of glaucoma-causing mutant myocilin thermal stability by chemical chaperones. *ACS Chem. Biol.* 5, 477-487. 10.1021/cb900282e PMC2874607 20334347

[DMM052821C11] Burns, J. N., Turnage, K. C., Walker, C. A. and Lieberman, R. L. (2011). The stability of myocilin olfactomedin domain variants provides new insight into glaucoma as a protein misfolding disorder. *Biochemistry* 50, 5824-5833. 10.1021/bi200231x PMC3128542 21612213

[DMM052821C12] Chen, S., Francioli, L. C., Goodrich, J. K., Collins, R. L., Kanai, M., Wang, Q., Alfoldi, J., Watts, N. A., Vittal, C., Gauthier, L. D. et al. (2024). A genomic mutational constraint map using variation in 76,156 human genomes. *Nature* 625, 92-100. 10.1038/s41586-023-06045-0 PMC11629659 38057664

[DMM052821C13] Cuervo, A. M., Stefanis, L., Fredenburg, R., Lansbury, P. T. and Sulzer, D. (2004). Impaired degradation of mutant α-synuclein by chaperone-mediated autophagy. *Science* 305, 1292-1295. 10.1126/science.1101738 15333840

[DMM052821C14] Donegan, R. K., Hill, S. E., Turnage, K. C., Orwig, S. D. and Lieberman, R. L. (2012). The glaucoma-associated olfactomedin domain of myocilin is a novel calcium binding protein. *J. Biol. Chem.* 287, 43370-43377. 10.1074/jbc.M112.408906 PMC3527924 23129764

[DMM052821C15] Donegan, R. K., Hill, S. E., Freeman, D. M., Nguyen, E., Orwig, S. D., Turnage, K. C. and Lieberman, R. L. (2015). Structural basis for misfolding in myocilin-associated glaucoma. *Hum. Mol. Genet.* 24, 2111-2124. 10.1093/hmg/ddu730 PMC4380063 25524706

[DMM052821C16] Elhusseiny, A. M. and Aref, A. A. (2025). Lifestyle modifications and environmental risk factors for glaucoma. *Curr. Opin Ophthalmol.* 36, 115-121. 10.1097/ICU.0000000000001098 39446700

[DMM052821C17] Emsley, P., Lohkamp, B., Scott, W. G. and Cowtan, K. (2010). Features and development of Coot. *Acta Crystallogr. D Biol. Crystallogr.* 66, 486-501. 10.1107/S0907444910007493 PMC2852313 20383002

[DMM052821C18] Ennis, S., Gibson, J., Griffiths, H., Bunyan, D., Cree, A. J., Robinson, D., Self, J., MacLeod, A. and Lotery, A. (2010). Prevalence of myocilin gene mutations in a novel UK cohort of POAG patients. *Eye* 24, 328-333. 10.1038/eye.2009.73 19407846

[DMM052821C19] Faralli, J. A., Desikan, H., Peotter, J., Kanneganti, N., Weinhaus, B., Filla, M. S. and Peters, D. M. (2019). Genomic/proteomic analyses of dexamethasone-treated human trabecular meshwork cells reveal a role for GULP1 and ABR in phagocytosis. *Mol. Vis.* 25, 237-254. PMC6706170 31516309

[DMM052821C20] Faucher, M., Anctil, J. L., Rodrigue, M. A., Duchesne, A., Bergeron, D., Blondeau, P., Cote, G., Dubois, S., Bergeron, J. and Arseneault, R. (2002). Founder TIGR/myocilin mutations for glaucoma in the Quebec population. *Hum. Mol. Genet.* 11, 2077-2090. 10.1093/hmg/11.18.2077 12189160

[DMM052821C21] Filla, M. S., Liu, X., Nguyen, T. D., Polansky, J. R., Brandt, C. R., Kaufman, P. L. and Peters, D. M. (2002). In vitro localization of TIGR/MYOC in trabecular meshwork extracellular matrix and binding to fibronectin. *Invest. Ophthalmol. Vis. Sci.* 43, 151-161. 11773026

[DMM052821C22] Fingert, J. H., Heon, E., Liebmann, J. M., Yamamoto, T., Craig, J. E., Rait, J., Kawase, K., Hoh, S. T., Buys, Y. M., Dickinson, J. et al. (1999). Analysis of myocilin mutations in 1703 glaucoma patients from five different populations. *Hum. Mol. Genet.* 8, 899-905. 10.1093/hmg/8.5.899 10196380

[DMM052821C23] Fini, M. E., Schwartz, S. G., Gao, X., Jeong, S., Patel, N., Itakura, T., Price, M. O., Price, F. W., Jr., Varma, R. and Stamer, W. D. (2017). Steroid-induced ocular hypertension/glaucoma: focus on pharmacogenomics and implications for precision medicine. *Prog. Retin. Eye Res.* 56, 58-83. 10.1016/j.preteyeres.2016.09.003 PMC5237612 27666015

[DMM052821C24] Flagg, M. P., Lam, B., Lam, D. K., Le, T. M., Kao, A., Slaiwa, Y. I. and Hampton, R. Y. (2023). Exploring the “misfolding problem” by systematic discovery and analysis of functional-but-degraded proteins. *Mol. Biol. Cell* 34, ar125. 10.1091/mbc.E23-06-0248 PMC10848938 37729018

[DMM052821C25] Gabelt, B. T. and Kaufman, P. L. (2005). Changes in aqueous humor dynamics with age and glaucoma. *Prog. Retin. Eye Res.* 24, 612-637. 10.1016/j.preteyeres.2004.10.003 15919228

[DMM052821C26] Gao, X. R., Chiariglione, M. and Arch, A. J. (2022). Whole-exome sequencing study identifies rare variants and genes associated with intraocular pressure and glaucoma. *Nat. Commun.* 13, 7376. 10.1038/s41467-022-35188-3 PMC9712679 36450729

[DMM052821C27] Ge, S. X., Jung, D. M. and Yao, R. A. (2020). ShinyGO: a graphical gene-set enrichment tool for animals and plants. *Bioinformatics* 36, 2628-2629. 10.1093/bioinformatics/btz931 PMC7178415 31882993

[DMM052821C28] Gidalevitz, T., Stevens, F. and Argon, Y. (2013). Orchestration of secretory protein folding by ER chaperones. *Biochim. Biophys. Acta* 1833, 2410-2424. 10.1016/j.bbamcr.2013.03.007 PMC3729627 23507200

[DMM052821C29] Gobeil, S., Letartre, L. and Raymond, V. (2006). Functional analysis of the glaucoma-causing TIGR/myocilin protein: integrity of amino-terminal coiled-coil regions and olfactomedin homology domain is essential for extracellular adhesion and secretion. *Exp. Eye Res.* 82, 1017-1029. 10.1016/j.exer.2005.11.002 16466712

[DMM052821C30] Gong, G., Kosoko-Lasaki, O., Haynatzki, G. R. and Wilson, M. R. (2004). Genetic dissection of myocilin glaucoma. *Hum. Mol. Genet.* 13, R91-R102. 10.1093/hmg/ddh074 14764620

[DMM052821C31] Gordon, M. O., Beiser, J. A., Brandt, J. D., Heuer, D. K., Higginbotham, E. J., Johnson, C. A., Keltner, J. L., Miller, J. P., Parrish, R. K., II and Wilson, M. R. (2002). The ocular hypertension treatment study: baseline factors that predict the onset of primary open-angle glaucoma. *Arch. Ophthalmol.* 120, 714-720. 10.1001/archopht.120.6.714 12049575

[DMM052821C32] Gould, D. B., Reedy, M., Wilson, L. A., Smith, R. S., Johnson, R. L. and John, S. W. (2006). Mutant myocilin nonsecretion in vivo is not sufficient to cause glaucoma. *Mol. Cell. Biol.* 26, 8427-8436. 10.1128/MCB.01127-06 PMC1636791 16954374

[DMM052821C33] Han, X., Souzeau, E., Ong, J. S., An, J., Siggs, O. M., Burdon, K. P., Best, S., Goldberg, I., Healey, P. R., Graham, S. L. et al. (2019). Myocilin gene Gln368Ter variant penetrance and association with glaucoma in population-based and registry-based studies. *JAMA Ophthalmol.* 137, 28-35. 10.1001/jamaophthalmol.2018.4477 PMC6439786 30267046

[DMM052821C34] Hill, S. E., Donegan, R. K. and Lieberman, R. L. (2014). The glaucoma-associated olfactomedin domain of myocilin forms polymorphic fibrils that are constrained by partial unfolding and peptide sequence. *J. Mol. Biol.* 426, 921-935. 10.1016/j.jmb.2013.12.002 PMC3946817 24333014

[DMM052821C35] Hipp, M. S., Kasturi, P. and Hartl, F. U. (2019). The proteostasis network and its decline in ageing. *Nat. Rev. Mol. Cell Biol.* 20, 421-435. 10.1038/s41580-019-0101-y 30733602

[DMM052821C36] Huard, D. J. E., Crowley, V. M., Du, Y., Cordova, R. A., Sun, Z., Tomlin, M. O., Dickey, C. A., Koren, J., III, Blair, L., Fu, H. et al. (2018). Trifunctional high-throughput screen identifies promising scaffold to inhibit Grp94 and treat myocilin-associated glaucoma. *ACS Chem. Biol.* 13, 933-941. 10.1021/acschembio.7b01083 PMC6195314 29402077

[DMM052821C37] Huard, D. J. E., Jonke, A. P., Torres, M. P. and Lieberman, R. L. (2019). Different Grp94 components interact transiently with the myocilin olfactomedin domain in vitro to enhance or retard its amyloid aggregation. *Sci. Rep.* 9, e12769. 10.1038/s41598-019-48751-8 PMC6726633 31484937

[DMM052821C38] Itakura, T., Peters, D. M. and Fini, M. E. (2015). Glaucomatous MYOC mutations activate the IL-1/NF-kappaB inflammatory stress response and the glaucoma marker SELE in trabecular meshwork cells. *Mol. Vis.* 21, 1071-1084. PMC4575906 26396484

[DMM052821C39] Jacobson, N., Andrews, M., Shepard, A. R., Nishimura, D., Searby, C., Fingert, J. H., Hageman, G., Mullins, R., Davidson, B. L. and Kwon, Y. H. (2001). Non-secretion of mutant proteins of the glaucoma gene myocilin in cultured trabecular meshwork cells and in aqueous humor. *Hum. Mol. Genet.* 10, 117-125. 10.1093/hmg/10.2.117 11152659

[DMM052821C40] Joe, M. K., Sohn, S., Hur, W., Moon, Y., Choi, Y. R. and Kee, C. (2003). Accumulation of mutant myocilins in ER leads to ER stress and potential cytotoxicity in human trabecular meshwork cells. *Biochem. Biophys. Res. Commun.* 312, 592-600. 10.1016/j.bbrc.2003.10.162 14680806

[DMM052821C41] Kanehisa, M., Furumichi, M., Sato, Y., Matsuura, Y. and Ishiguro-Watanabe, M. (2025). KEGG: biological systems database as a model of the real world. *Nucleic Acids Res.* 53, D672-D677. 10.1093/nar/gkae909 PMC11701520 39417505

[DMM052821C42] Keller, K. E., Bhattacharya, S. K., Borras, T., Brunner, T. M., Chansangpetch, S., Clark, A. F., Dismuke, W. M., Du, Y., Elliott, M. H., Ethier, C. R. et al. (2018). Consensus recommendations for trabecular meshwork cell isolation, characterization and culture. *Exp. Eye Res.* 171, 164-173. 10.1016/j.exer.2018.03.001 PMC6042513 29526795

[DMM052821C43] Landrum, M. J., Lee, J. M., Benson, M., Brown, G. R., Chao, C., Chitipiralla, S., Gu, B., Hart, J., Hoffman, D., Jang, W. et al. (2018). ClinVar: improving access to variant interpretations and supporting evidence. *Nucleic Acids Res**.* 46, D1062-D1067. 10.1093/nar/gkx1153 PMC5753237 29165669

[DMM052821C44] Lieberman, R. L. and Ma, M. T. (2021). Molecular insights into myocilin and its glaucoma-causing misfolded olfactomedin domain variants. *Acc. Chem. Res.* 54, 2205-2215. 10.1021/acs.accounts.1c00060 PMC8771271 33847483

[DMM052821C45] Liton, P. B., Challa, P., Stinnett, S., Luna, C., Epstein, D. L. and Gonzalez, P. (2005). Cellular senescence in the glaucomatous outflow pathway. *Exp. Gerentol.* 40, 745-748. 10.1016/j.exger.2005.06.005 PMC3152456 16051457

[DMM052821C46] Liu, T. and He, X. G. (2007). [Meta-analysis on the association of Myocilin Q368X mutation and primary open angle glaucoma]. *Zhonghua Yan Ke Za Zhi* 43, 361-366. PMID: 17605937; 10.3760/j.issn:0412-4081.2007.04.015 17605937

[DMM052821C47] Liu, Y. and Vollrath, D. (2004). Reversal of mutant myocilin non-secretion and cell killing: implications for glaucoma. *Hum. Mol. Genet.* 13, 1193-1204. 10.1093/hmg/ddh128 15069026

[DMM052821C48] Ma, M. T., Qerqez, A. N., Hill, K. R., Azouz, L. R., Youngblood, H. A., Hill, S. E., Ku, Y., Peters, D. M., Maynard, J. A. and Lieberman, R. L. (2025). Antibody-mediated clearance of an ER-resident aggregate that causes glaucoma. *PNAS Nexus* 4, pgae556. 10.1093/pnasnexus/pgae556 PMC11670252 39726989

[DMM052821C49] Manolio, T. A., Collins, F. S., Cox, N. J., Goldstein, D. B., Hindorff, L. A., Hunter, D. J., McCarthy, M. I., Ramos, E. M., Cardon, L. R., Chakravarti, A. et al. (2009). Finding the missing heritability of complex diseases. *Nature* 461, 747-753. 10.1038/nature08494 PMC2831613 19812666

[DMM052821C50] Masters, J. R., Thomson, J. A., Daly-Burns, B., Reid, Y. A., Dirks, W. G., Packer, P., Toji, L. H., Ohno, T., Tanabe, H., Arlett, C. F. et al. (2001). Short tandem repeat profiling provides an international reference standard for human cell lines. *Proc. Natl. Acad. Sci. USA* 98, 8012-8017. 10.1073/pnas.121616198 PMC35459 11416159

[DMM052821C51] McCoy, A. J. (2007). Solving structures of protein complexes by molecular replacement with Phaser. *Acta Crystallogr. D Biol. Crystallogr.* 63, 32-41. 10.1107/S0907444906045975 PMC2483468 17164524

[DMM052821C52] McPherson, A. and Gavira, J. A. (2014). Introduction to protein crystallization. *Acta Crystallogr. F* 70, 2-20. 10.1107/S2053230X13033141 PMC3943105 24419610

[DMM052821C53] Molinari, M. (2021). ER-phagy responses in yeast, plants, and mammalian cells and their crosstalk with UPR and ERAD. *Dev. Cell* 56, 949-966. 10.1016/j.devcel.2021.03.005 33765438

[DMM052821C54] Murnane, J. P., Fuller, L. F. and Painter, R. B. (1985). Establishment and characterization of a permanent pSV ori--transformed ataxia-telangiectasia cell line. *Exp. Cell Res.* 158, 119-126. 10.1016/0014-4827(85)90437-9 2987007

[DMM052821C55] Nag, A., Lu, H., Arno, M., Iglesias, A. I., Bonnemaijer, P., Broer, L., Uitterlinden, A. G., Klaver, C. C., van Duijn, C., Hysi, P. G. et al. (2017). Evaluation of the myocilin mutation Gln368Stop demonstrates reduced penetrance for glaucoma in european populations. *Ophthalmology* 124, 547-553. 10.1016/j.ophtha.2016.11.018 28038983

[DMM052821C56] Nguyen, T. D., Chen, P., Huang, W. D., Chen, H., Johnson, D. and Polansky, J. R. (1998). Gene structure and properties of TIGR, an olfactomedin-related glycoprotein cloned from glucocorticoid-induced trabecular meshwork cells. *J. Biol. Chem.* 273, 6341-6350. 10.1074/jbc.273.11.6341 9497363

[DMM052821C57] O'Gorman, L., Cree, A. J., Ward, D., Griffiths, H. L., Sood, R., Denniston, A. K., Self, J. E., Ennis, S., Lotery, A. J. and Gibson, J. (2019). Comprehensive sequencing of the myocilin gene in a selected cohort of severe primary open-angle glaucoma patients. *Sci. Rep.* 9, 3100. 10.1038/s41598-019-38760-y PMC6395666 30816137

[DMM052821C58] Orwig, S. D., Perry, C. W., Kim, L. Y., Turnage, K. C., Zhang, R., Vollrath, D., Schmidt-Krey, I. and Lieberman, R. L. (2012). Amyloid fibril formation by the glaucoma-associated olfactomedin domain of myocilin. *J. Mol. Biol.* 421, 242-255. 10.1016/j.jmb.2011.12.016 PMC3323732 22197377

[DMM052821C59] Otwinowski, Z. and Minor, W. (1997). Processing of X-ray diffraction data collected in oscillation mode. *Methods Enzymol.* 276, 307-326. 10.1016/S0076-6879(97)76066-X 27754618

[DMM052821C60] Overby, D. R. and Clark, A. F. (2015). Animal models of glucocorticoid-induced glaucoma. *Exp. Eye Res.* 141, 15-22. 10.1016/j.exer.2015.06.002 PMC4628841 26051991

[DMM052821C61] Patel, G. C., Phan, T. N., Maddineni, P., Kasetti, R. B., Millar, J. C., Clark, A. F. and Zode, G. S. (2017). Dexamethasone-induced ocular hypertension in mice: effects of myocilin and route of administration. *Am. J. Pathol.* 187, 713-723. 10.1016/j.ajpath.2016.12.003 PMC5397678 28167045

[DMM052821C62] Patterson-Orazem, A. C., Hill, S. E., Wang, Y., Dominic, I. M., Hall, C. K. and Lieberman, R. L. (2019). Differential misfolding properties of glaucoma-associated olfactomedin domains from humans and mice. *Biochemistry* 58, 1718-1727. 10.1021/acs.biochem.8b01309 PMC6815101 30802039

[DMM052821C63] Polansky, J. R., Weinreb, R. N., Baxter, J. D. and Alvarado, J. (1979). Human trabecular cells. I. Establishment in tissue culture and growth characteristics. *Invest. Ophthalmol. Vis. Sci.* 18, 1043-1049. 383640

[DMM052821C64] Polansky, J. R., Wood, I. S., Maglio, M. T. and Alvarado, J. A. (1984). Trabecular meshwork cell culture in glaucoma research: evaluation of biological activity and structural properties of human trabecular cells in vitro. *Ophthalmology* 91, 580-595. 10.1016/S0161-6420(84)34241-5 6540429

[DMM052821C65] Qiu, Y., Shen, X., Shyam, R., Yue, B. Y. J. T. and Ying, H. (2014). Cellular processing of myocilin. *PLOS ONE* 9, e92845. 10.1371/journal.pone.0092845 PMC3986080 24732711

[DMM052821C66] Resch, Z. T. and Fautsch, M. P. (2009). Glaucoma-associated myocilin: a better understanding but much more to learn. *Exp. Eye Res.* 88, 704-712. 10.1016/j.exer.2008.08.011 PMC2682697 18804106

[DMM052821C67] Saccuzzo, E. G., Mebrat, M. D., Scelsi, H. F., Kim, M., Ma, M. T., Su, X., Hill, S. E., Rheaume, E., Li, R., Torres, M. P. et al. (2024). Competition between inside-out unfolding and pathogenic aggregation in an amyloid-forming beta-propeller. *Nat. Commun.* 15, 155. 10.1038/s41467-023-44479-2 PMC10762032 38168102

[DMM052821C68] Santra, M., Dill, K. A. and de Graff, A. M. R. (2019). Proteostasis collapse is a driver of cell aging and death. *Proc. Natl. Acad. Sci. U.S.A.* 116, 22173-22178. 10.1073/pnas.1906592116 PMC6825304 31619571

[DMM052821C69] Scelsi, H. F., Hill, K. R., Barlow, B. M., Martin, M. D. and Lieberman, R. L. (2023). Quantitative differentiation of benign and misfolded glaucoma-causing myocilin variants on the basis of protein thermal stability. *Dis. Model. Mech.* 16, dmm049816. 10.1242/dmm.049816 PMC9844228 36579626

[DMM052821C70] Scelsi, H. F., Close, E. G. S., Huard, D. J. E., Dunn, E., Bogdanovic, N., Mudiyanselage, S. H. W., Grant, A., Stagg, S. M., Schmidt-Krey, I., Van Horn, W. D. et al. (2025). Detection of non-native species formed during fibrillization of the myocilin olfactomedin domain. *Protein Sci.* 34, e70063. 10.1002/pro.70063 PMC11912428 40095382

[DMM052821C71] Schneider, C. A., Rasband, W. S. and Eliceiri, K. W. (2012). NIH Image to ImageJ: 25 years of image analysis. *Nat. Methods* 9, 671-675. 10.1038/nmeth.2089 PMC5554542 22930834

[DMM052821C72] Shim, M. S. and Liton, P. B. (2022). The physiological and pathophysiological roles of the autophagy lysosomal system in the conventional aqueous humor outflow pathway: more than cellular clean up. *Prog. Retin. Eye Res.* 90, 101064. 10.1016/j.preteyeres.2022.101064 PMC9464695 35370083

[DMM052821C73] Shin, K. J., Wall, E. A., Zavzavadjian, J. R., Santat, L. A., Liu, J., Hwang, J. I., Rebres, R., Roach, T., Seaman, W., Simon, M. I. et al. (2006). A single lentiviral vector platform for microRNA-based conditional RNA interference and coordinated transgene expression. *Proc. Natl. Acad. Sci. USA* 103, 13759-13764. 10.1073/pnas.0606179103 PMC1557799 16945906

[DMM052821C74] Stone, E. M., Fingert, J. H., Alward, W. L. M., Nguyen, T. D., Polansky, J. R., Sunden, S. L. F., Nishimura, D., Clark, A. F., Nystuen, A., Nichols, B. E. et al. (1997). Identification of a gene that causes primary open angle glaucoma. *Science* 275, 668-670. 10.1126/science.275.5300.668 9005853

[DMM052821C75] Stothert, A. R., Suntharalingam, A., Huard, D. J., Fontaine, S. N., Crowley, V. M., Mishra, S., Blagg, B. S., Lieberman, R. L. and Dickey, C. A. (2014). Exploiting the interaction between Grp94 and aggregated myocilin to treat glaucoma. *Hum. Mol. Genet.* 23, 6470-6480. 10.1093/hmg/ddu367 PMC4240202 25027323

[DMM052821C76] Stothert, A. R., Suntharalingam, A., Tang, X., Crowley, V. M., Mishra, S. J., Webster, J. M., Nordhues, B. A., Huard, D. J. E., Passaglia, C. L., Lieberman, R. L. et al. (2017). Isoform-selective Hsp90 inhibition rescues model of hereditary open-angle glaucoma. *Sci. Rep.* 7, 17951. 10.1038/s41598-017-18344-4 PMC5738387 29263415

[DMM052821C77] Sun, Y., Chen, A., Zou, M., Zhang, Y., Jin, L., Li, Y., Zheng, D., Jin, G. and Congdon, N. (2022). Time trends, associations and prevalence of blindness and vision loss due to glaucoma: an analysis of observational data from the Global Burden of Disease Study 2017. *BMJ Open* 12, e053805. 10.1136/bmjopen-2021-053805

[DMM052821C78] Suntharalingam, A., Abisambra, J. F., O'Leary, J. C., III, Koren, J., III, Zhang, B., Joe, M. K., Blair, L. J., Hill, S. E., Jinwal, U. K., Cockman, M. et al. (2012). Glucose-regulated protein 94 triage of mutant myocilin through endoplasmic reticulum-associated degradation subverts a more efficient autophagic clearance mechanism. *J. Biol. Chem.* 287, 40661-40669. 10.1074/jbc.M112.384800 PMC3504779 23035116

[DMM052821C79] Walczak, M. and Martens, S. (2013). Dissecting the role of the Atg12-Atg5-Atg16 complex during autophagosome formation. *Autophagy* 9, 424-425. 10.4161/auto.22931 PMC3590266 23321721

[DMM052821C80] Wang, E. (1995). Senescent human fibroblasts resist programmed cell death, and failure to suppress bcl2 is involved. *Cancer Res.* 55, 2284-2292. 7757977

[DMM052821C81] Wang, Z. and Moult, J. (2001). SNPs, protein structure, and disease. *Hum. Mutat.* 17, 263-270. 10.1002/humu.22 11295823

[DMM052821C82] Wiggs, J. L., Howell, G. R., Linkroum, K., Abdrabou, W., Hodges, E., Braine, C. E., Pasquale, L. R., Hannon, G. J., Haines, J. L. and John, S. W. (2013). Variations in COL15A1 and COL18A1 influence age of onset of primary open angle glaucoma. *Clin. Genet.* 84, 167-174. 10.1111/cge.12176 PMC3771394 23621901

[DMM052821C83] Willoughby, C. E., Chan, L. L., Herd, S., Billingsley, G., Noordeh, N., Levin, A. V., Buys, Y., Trope, G., Sarfarazi, M. and Heon, E. (2004). Defining the pathogenicity of optineurin in juvenile open-angle glaucoma. *Invest. Ophthalmol. Vis. Sci.* 45, 3122-3130. 10.1167/iovs.04-0107 15326130

[DMM052821C84] Yam, G. H.-F., Gaplovska-Kysela, K., Zuber, C. and Roth, J. (2007). Aggregated myocilin induces russell bodies and causes apoptosis: implications for the pathogenesis of myocilin-caused primary open-angle glaucoma. *Am. J. Pathol.* 170, 100-109. 10.2353/ajpath.2007.060806 PMC1762699 17200186

[DMM052821C85] Yan, X., Wu, S., Liu, Q., Teng, Y., Wang, N. and Zhang, J. (2024). The S341P mutant MYOC renders the trabecular meshwork sensitive to cyclic mechanical stretch. *Heliyon* 10, e37137. 10.1016/j.heliyon.2024.e37137 PMC11402775 39286096

[DMM052821C86] Young, T. K., Souzeau, E., Liu, L., Kearns, L. S., Burdon, K. P., Craig, J. E. and Ruddle, J. B. (2012). Compound heterozygote myocilin mutations in a pedigree with high prevalence of primary open-angle glaucoma. *Mol. Vis.* 18, 3064-3069. PMC3538040 23304066

[DMM052821C87] Yu, M., Liu, S., Zhang, C. and Yang, W. (2025). Revisiting ROS: duality in organ aging progression. *Mol. Biol. Rep.* 53, 202. 10.1007/s11033-025-11342-0 41396329

[DMM052821C88] Zhou, Z. and Vollrath, D. (1999). A cellular assay distinguishes normal and mutant TIGR/myocilin protein. *Hum. Mol. Genet.* 8, 2221-2228. 10.1093/hmg/8.12.2221 10545602

