# Supplementary material for: Trabecular meshwork cellular mis-trafficking of near-native and premature stop variants in glaucoma-relevant myocilin
Source: Dis Model Mech. 2026 Oct 30;19(10):dmm052821. doi: 10.1242/dmm.052821 (PMC13750402; doi:10.1242/dmm.052821)
Supplement: Supplementary file 1 [file dmm-19-052821-s1.pdf]

Table S1. Population-based and clinical data for Q368X and A427T. Data referenced from gnomAD (v4.1.0), ClinVar (Oct 26, 2025 update), and myocilin.com (Sep 28, 2025 update).

**ClinVar and gnomAD Data**	**Genetic Ancestry**	**Allele frequency (count)**	**Population & Reference**	**(Number of) Individual diagnoses and age at diagnosis when available**
**A427T**<br>Accession: **VCV001686783.3**<br>Classification: **Uncertain Significance**<br>Total Frequency (Allele count): **0.00002230 (36)**	European (non-Finnish)	0.00001610 (19)	**Caucasian**<br>ref (Faucher et al., 2002)	(1) POAG 73yo<br>(1) NTG 68yo<br>(1) ACG 77yo<br>(3) Unaffected 40.5yo
			**Spanish**<br>ref (Milla et al., 2013)	(1) NTG
	South Asian	0.0001427 (13)	**Indian**<br>ref (Bhattacharjee et al., 2007)	(1) POAG, PD
			**Indian**<br>ref (Banerjee et al., 2012)	(2) POAG<br>76yo; 46yo
	Admixed American	0.00003334 (2)	n/a	n/a
	Other (remaining)	0.00003201 (2)	n/a	n/a
**Q368X**<br>Accession: **VCV000007949.64**<br>Classification: **Pathogenic**<br>Total Frequency (Allele count): 0.001159 (1870)	European	0.003296 (211)	**Finnish**<br>ref (Liuska et al., 2023)	(1) JOAG 37yo
	European (non-Finnish)	0.001333 (1573)	**Sweden**<br>ref (Matafsti et al., 2001)	(5) POAG<br>53.4±20.1yo
			**French**<br>ref (Melki et al., 2003)	(12) POAG
			**English Canadian**<br>ref (Willoughby et al., 2004)	(1) JOAG 39yo<br>(3) 42.3±9.5yo<br>*di-heterozygous $MYOC^{K398R}$ and $CYP1B1^{L41L}$

			**Dutch**<br>ref (Hogewind et al., 2010)	(2) POAG<br>33.5±3.5yo
			**Australian**<br>ref (Bennett et al., 2007)	(14) Unaffected<br>21.1±11.6yo
			**Australian**<br>ref (Craig et al., 2001)	(29) POAG or OHT<br>52.4± 2.9yo
			ref (Sherwin et al., 2009)	(9) POAG 54.3yo
			**Brazilian**<br>ref (Svidnicki et al., 2018)	(2) JOAG 34.5±5yo
			**Italian**<br>ref (Angius et al., 2000)	(24) POAG<br>54±11.6yo<br>(7) OHT<br>(5) Unaffected
			**Scottish**<br>ref (Cobb et al., 2002)	(5) 44.2±8yo<br>(4) OHT<br>(1) Unaffected<br>(1) POAG
			**Spanish**<br>ref (Milla et al., 2013)	(2) POAG<br>(1) OHT<br>(1) Unaffected
			**Spanish**<br>ref (Vazquez et al., 2000)	(1) POAG 72yo
			**Spanish**<br>ref (Lopez-Martinez et al., 2007)	(1) POAG 56yo
			**Caucasian**<br>ref (Aldred et al., 2004)	(6) POAG
			**Caucasian**<br>ref (Allingham et al., 1998)	(16) 55.6±14.9yo
			**Caucasian**<br>ref (Alward et al., 2019)	(11) NTG

Disease Models & Mechanisms • Supplementary information

			**Caucasian** ref (Alward et al., 2002)	(12) total:<br>(9) POAG<br>(1) JOAG<br>(1) OHT<br>(1) pigment dispersion syndrome
			**Caucasian** ref (Alward et al., 1998)	(25) POAG 59yo<br>(1) Unaffected
			**Caucasian** ref (Baird et al., 2005a)	(9) POAG
			**Caucasian** ref (Baird et al., 2005b)	(2) Unaffected
			**Caucasian** ref (Baird et al., 2005c)	(15) POAG
			**Caucasian** ref (Baird et al., 2003)	(38) POAG
			**Caucasian** ref (Ennis et al., 2010)	(5) POAG 57.8±12.6yo
			**Caucasian** ref (Faucher et al., 2002)	(8) POAG or ACG 56±14yo<br>(1) Unaffected 71yo<br>(6) POAG 55.3yo (French Canadian)
			**Caucasian** ref (Fingert et al., 2001)	(1) OHT
			**Caucasian** ref (Graul et al., 2002)	(22) 52.1±3.1yo
			**Caucasian** ref (Hewitt et al., 2006)	(1) Unaffected 39yo
			**Caucasian** ref (Jansson et al., 2003)	(2) POAG<br>(1) Unaffected 82yo

Disease Models & Mechanisms • Supplementary information

			**Caucasian** ref (Mardin et al., 1999)	(1) NTG 38yo
			**Caucasian** ref (Michels-Rautenstrauss et al., 2002)	(1) POAG<br>(1) OHT<br>(1) NTG
			**Caucasian** ref (O'Gorman et al., 2019)	(7) POAG 69.6±15.3yo<br>*di-heterozygous $MYOC^{T419A}$
			**Caucasian** ref (Rakhmanov et al., 2005)	(2) POAG
			**Caucasian** ref (Shimizu et al., 2000)	(8) POAG 37yo<br>(2) POAG 39±2.8yo
			**Caucasian** ref (Stone et al., 1997)	(1) Unaffected
			**Caucasian** ref (Young et al., 2012)	(3) POAG 21yo<br>(1) *Heterozygous $MYOC^{T377M}$
			**Mixed** (Villalba et al., 2022)	(1) PCG (Caucasian)
			**Mixed** ref (Fingert et al., 1999)	(27) POAG<br>1 Caucasian, 1 African American
			**Mixed** ref (Souzeau et al., 2013)	(28) POAG 54±15.9yo
			**Mixed** ref (Vincent et al., 2002)	(1) POAG 39yo English
	African/African American	0.0002934 (22)	**African-American** ref (Liu et al., 2012)	(1) POAG 56yo
	Admixed American	0.0002000 (12)	**Mixed** ref (Williams-Lyn et al., 2000)	(3) POAG 47±11.4yo

Disease Models & Mechanisms • Supplementary information

			**Mixed**<br>ref (Wiggs et al., 1998)	(3) POAG<br>60±15.7yo
			**Mixed**<br>ref (Wiggs et al., 2013)	(3) POAG<br>(2) POAG<br>*Heterozygous *COL18A1*^{A1381T}
			**Indian**<br>ref (Banerjee et al., 2012)	(3) POAG<br>55.3±4yo
			**Indian**<br>ref (Bhattacharjee et al., 2007)	(2) POAG<br>58.5±5yo
			**Chinese**<br>ref (Liu et al., 2020)	(1) POAG 48yo
	South Asian	0.0001318 (12)		
	Other (remaining)	0.0006400 (40)		

Abbreviations: POAG, primary open-angle glaucoma; NTG, normal tension glaucoma; ACG, angle closure glaucoma; PD, Parkinson's disease; PCG, primary congenital glaucoma; () #, number of affected individuals and mean age of diagnosis if available; *, heterozygous mutations found.

Disease Models & Mechanisms • Supplementary information

Table S2. X-ray data collection and refinement statistics. Statistics for the highest-resolution shell are shown in parentheses.

	OLF^{A427T} (PDB ID: 9DRW)
**Wavelength**	1
**Resolution range**	58.6 - 1.45 (1.50 - 1.45)
**Space group**	C 1 2 1
**Unit cell**	140.93 47.81 149.99 90 107.71 90
**Total reflections**	1084929 (73615)
**Unique reflections**	160850 (12829)
**Multiplicity**	6.7 (5.7)
**Completeness (%)**	95.1 (76.5)
**Mean I/sigma(I)**	16.2 (4.45)
**Wilson B-factor**	14.7
**R-merge**	0.0641 (0.538)
**R-meas**	0.0695 (0.593)
**R-pim**	0.0266 (0.241)
**CC1/2**	0.999 (0.912)
**CC***	1 (0.977)
**Reflections used in refinement**	160737 (12824)
**Reflections used for R-free**	1999 (159)
**R-work**	0.146 (0.215)
**R-free**	0.188 (0.291)
**CC(work)**	0.974 (0.946)
**CC(free)**	0.963 (0.921)
**Number of non-hydrogen atoms**	9118
**macromolecules**	8345
**ligands**	14
**solvent**	759
**Protein residues**	1029
**RMS(bonds)**	0.004
**RMS(angles)**	0.81
**Ramachandran favored (%)**	96.5
**Ramachandran allowed (%)**	3.46
**Ramachandran outliers (%)**	0.00
**Rotamer outliers (%)**	0.00
**Clashscore**	3.53
**Average B-factor**	21.2
**macromolecules**	20.0
**ligands**	32.4
**solvent**	33.6

Disease Models & Mechanisms • Supplementary information

Table S3. KEGG pathway enrichment of TM-1 cells expressing myocilin

A. WT (vs no induction)

Enrichment FDR	# of Genes/ # in Pathway	Fold Enrichment	KEGG Pathway	Genes
Upregulated				
3.59E-13	54/132	3.1	Path:hsa04142 Lysosome	NPC2 AP4S1 TPP1 CLN5 CLTA CLTC HGSNAT CTNS CTSD CTSH DNASE2 ABCA2 GGA2 ATP6V0A2 FUCA2 GAA SLC17A5 GNS GUSB HEXA HEXB IDUA IGF2R LAMP1 M6PR ARSA MANBA ASAH1 NAGA NAGLU NPC1 ACP2 ATP6V0B ATP6V0A1 ATP6AP1 CTSA LAPTM4B PPT1 PSAP MCOLN1 SORT1 SGSH SMPD1 GNPTAB GNPTG HYAL2 CTSF CD164 AP1S2 AP1G2 AP4M1 SCARB2 LITAF CD63
2.03E-10	58/169	2.6	Path:hsa04141 Protein processing in endoplasmic reticulum	PDIA6 BCAP31 STUB1 SEC23B HSPH1 OS9 LMAN2 ERP29 EDEM2 SEC63 MAN1B1 TRAM1L1 DAD1 DDIT3 DDOST EIF2S1 STT3B GANAB TRAM1 HSPBP1 PPP1R15A SVIP SEC61A1 UBQLN1 DNAJB2 DNAJA1 HSPA5 HSPA6 HSPA8 DNAJB1 STT3A ATF4 NFE2L2 DERL2 UBXN1 DNAJB11 SELENOS DNAJC3 BAG1 RAD23A BCL2 RPN1 SEC13 DNAJC1 SIL1 SSR1 SSR2 SSR3 SSR4 SEC62 XBP1 TUSC3 UBXN8 CALR SYVN1 RNF185 EIF2AK3 BAG2
1.79E-07	24/50	3.6	Path:hsa00510 N-Glycan biosynthesis	MAN1B1 DAD1 DDOST DPAGT1 STT3B DOLK GANAB ALG5 STT3A MAN2A2 MAN2A1 MGAT1 ALG11 DPM3 ALG1 DOLPP1 RPN1 ALG8 ALG12 TUSC3 ST6GAL2 ALG10 ALG2 DPM1
2.97E-04	16/39	3.1	Path:hsa00513 Various types of N-glycan biosynthesis	MAN1B1 DAD1 DDOST STT3B HEXA HEXB STT3A MAN2A2 MAN2A1 MGAT1 ALG11 ALG1 RPN1 ALG12 TUSC3 ALG2
Downregulated				
2.78E-08	48/142	2.5	Path:hsa04120 Ubiquitin mediated proteolysis	SAE1 HUWE1 WWP1 WWP2 FBXW11 MGRN1 ANAPC15 HERC4 UBE2S PRPF19 ANAPC4 MAP3K1 NEDD4 FZR1 UBR5 UBE2J1 UBE2R2 FBXW7 UBE2Q1 BIRC6 UBE2O COP1 SKP2 UBB UBA1 UBE2D1 UBE2D2 UBE2G1 UBE2I VHL CUL5 CUL4B CUL4A CUL2 CUL1 CBL HERC1 BTRC UBE3B SOCS3 KLHL13 UBE2M UBE2Q2 TRIP12 UBE3C CDC20 CDC27 CDC34

Disease Models & Mechanisms • Supplementary information

B. Y437H vs WT

Enrichment FDR	# of Genes/ # in Pathway	Fold Enrichment	KEGG Pathway	Genes
Upregulated				
8.99E-06	52/142	2.0	Path:hsa04120 Ubiquitin mediated proteolysis	HUWE1 WWP2 UBE2J2 ANAPC16 DDB1 MGRN1 NEDD4L ANAPC15 FBXW8 PRPF19 ANAPC4 UBE2K BIRC2 XIAP MDM2 MAP3K1 MID1 NEDD4 ANAPC5 ANAPC7 UBE2J1 UBE2R2 UBA6 FBXW7 KLHL9 BIRC6 RPS27A COP1 SKP1 SKP2 UBB UBA1 UBE2A UBE2D3 UBE2E1 UBE2G1 UBE2G2 UBE2I UBE2L3 UBE2N UBE3A CUL5 CUL4B CUL2 CDC16 BTRC UBE3B UBA3 PIAS2 TRIP12 UBE4A CDC20
Downregulated				
7.85E-12	78/169	2.2	Path:hsa04141 Protein processing in endoplasmic reticulum	PREB PDIA6 STUB1 UBE4B MARCHF6 SEC23B SEC23A SEC24A OS9 LMAN2 ERP29 MAN1B1 DAD1 DDIT3 DDOST SEC31A UBXN4 TRAM1 SEC61G HSPBP1 PPP1R15A SVIP FBXO2 AMFR EIF2AK1 UBQLN1 ERO1A DNAJB2 HSPA5 HSPA6 HSPA8 HSP90AA1 DNAJB1 MAN1A1 MAP3K5 ATF4 NFE2L2 P4HB UBXN1 UBE2D4 DNAJB11 DNAJC10 DNAJB12 NPLOC4 UGGT2 SELENOS PRKCSH MAP2K7 DNAJC3 ERO1B UGGT1 UBQLN4 MAN1C1 BAX RAD23A RAD23B RPN1 RPN2 SEL1L SSR1 SSR2 SSR4 SEC62 TRAF2 UBE2D2 XBP1 DERL1 TUSC3 UBXN6 CALR SYVN1 CUL1 RNF185 EIF2AK3 PDIA4 SEC24C HERPUD1 SEC24D
6.23E-09	60/132	2.2	Path:hsa04142 Lysosome	AP3S2 NPC2 AP3M2 AP3S1 TPP1 CLTC AP1S3 NCOA7 CTNS CTSD CTSH CTSL DNASE2 ABCA2 GGA3 DMXL2 ATP6V0A2 ATP6V0D2 FUCA1 GAA SLC17A5 AP3M1 GLB1 GNS GUSB HEXA HEXB IGF2R LAMP2 LIPA M6PR ARSA MAN2B1 MANBA ASAH1 NAGA NAGLU NPC1 ATP6V1H ACP2 ATP6V0A1 ATP6AP1 CTSA LAPTM4B PPT1 PSAP MCOLN1 SGSH SMPD1 GNPTAB CLTCL1 GNPTG AP3B1 HYAL2 CTSF AP1M1 AP3D1 AP4M1 SCARB2 LITAF
4.65E-06	56/142	1.91	Path:hsa04120 Ubiquitin mediated proteolysis	SAE1 STUB1 UBE4B UBE2E3 WWP1 UBE2C ERCC8 UBE2F UBOX5 TRIM32 FBXW11 CDC26 FBXO2 FBXO4 UBE2S ANAPC2 BIRC3 TRIM37 FZR1 UBR5 ANAPC11 PIAS4 UBE2D4 UBE2W UBE2O ANAPC1 SMURF2 SIAH1 UBE2Z BRCA1 ELOC TRAF6 UBA52 UBC UBA7 UBE2B UBE2D2 UBE2E2 UBE2H VHL ITCH SYVN1 CUL1 PIAS1 SOCS1 CBLB CDC23 SOCS3 UBE2M UBE2L6 UBE2Q2 UBE3C KEAP1 CUL7 CDC27 CDC34

Disease Models & Mechanisms • Supplementary information

C. Q368X vs WT

Enrichment FDR	# of Genes/ # in Pathway	Fold Enrichment	KEGG Pathway	Genes
Upregulated				
1.46E-08	50/142	2.6	Path:hsa04120 Ubiquitin mediated proteolysis	HUWE1 PIAS3 WWP2 ANAPC16 UBE2F FBXW11 MGRN1 HERC4 UBE2S ANAPC2 ANAPC4 XIAP MAP3K1 FZR1 ANAPC5 ANAPC7 PIAS4 PML BIRC6 UBE2O SKP1 SKP2 UBE2Z BRCA1 UBB UBA1 UBE2A UBE2D1 UBE2D2 UBE2D3 UBE2E1 UBE2G1 UBE2I UBE2N CUL5 CUL3 CUL2 CUL1 HERC1 BTRC SOCS3 KLHL13 UBA3 UBE2Q2 TRIP12 UBE4A UBE3C KEAP1 CDC20 CDC27
Downregulated				
2.80E-10	47/132	3.0	Path:hsa04142 Lysosome	NPC2 AP3M2 TPP1 CLN5 NCOA7 HGSNAT CTNS CTSD CTSH DMXL1 DNASE2 ABCA2 ATP6V0A2 GAA GALC GGA1 SLC17A5 GLB1 GNS GUSB HEXA IDUA IGF2R LAMP1 LAMP2 M6PR ARSA MAN2B1 MANBA NAGA NAGLU NPC1 ACP2 ATP6AP1 CTSA PPT1 PSAP MCOLN1 SGSH GNPTAB CLTCL1 GNPTG CTSF AP1G2 AP4M1 SCARB2 LITAF
3.31E-08	50/169	2.5	Path:hsa04141 Protein processing in endoplasmic reticulum	PREB PDIA6 SEC23A OS9 ERP29 EDEM2 SEC63 MAN1B1 TRAM1L1 DDIT3 DDOST SEC31A GANAB TRAM1 DNAJB2 HSPA5 HSPA6 HSPA8 STT3A ATF4 NFE2L2 P4HB DERL2 UBXN1 DNAJB11 UGGT2 SELENOS DNAJC3 ERO1B MAN1C1 BAG1 RAD23A BCL2 RPN1 SEL1L DNAJC1 SIL1 HSP90B1 UFD1 XBP1 TUSC3 UBXN6 CALR SYVN1 RNF185 EIF2AK3 PDIA4 EDEM1 HERPUD1 SEC24D

Disease Models & Mechanisms • Supplementary information

D. A427T vs WT

Enrichment FDR	# of Genes/ # in Pathway	Fold Enrichment	KEGG Pathway	Genes
Upregulated				
5.01E-15	30/46	5.2	Path:hsa03050 Proteasome	PSMD14 ADRM1 PSME4 POMP PSMA3 PSMA4 PSMA5 PSMA7 PSMB1 PSMB2 PSMB4 PSMB5 PSMB6 PSMB7 PSMC1 PSMC2 PSMC3 PSMC5 PSMC6 PSMD1 PSMD2 PSMD3 PSMD4 PSMD7 PSMD8 PSMD11 PSMD12 PSMD13 SEM1 PSMF1
1.71E-12	53/142	2.9	Path:hsa04120 Ubiquitin mediated proteolysis	SAE1 HUWE1 UBE2E3 WWP1 UBE2C UBE2J2 ANAPC16 FBXW11 MGRN1 NEDD4L ANAPC15 HERC4 UBE2S PRPF19 MAP3K1 MID1 NEDD4 FZR1 UBR5 ANAPC7 UBE2J1 UBA6 UBE2W BIRC6 RPS27A UBE2O ANAPC1 SMURF2 SKP2 ELOC ELOB UBB UBA1 UBE2D1 UBE2D2 UBE2D3 UBE2G1 UBE2I UBE2N UBE3A CUL5 CUL2 CUL1 HERC1 BTRC SOCS3 UBE2M TRIP12 UBE3C KEAP1 CDC20 CDC27 CDC34
Downregulated				
2.80E-10	47/132	3.0	Path:hsa04142 Lysosome	NPC2 AP3M2 TPP1 CLN5 NCOA7 HGSNAT CTNS CTSD CTSH DMXL1 DNASE2 ABCA2 ATP6V0A2 GAA GALC GGA1 SLC17A5 GLB1 GNS GUSB HEXA IDUA IGF2R LAMP1 LAMP2 M6PR ARSA MAN2B1 MANBA NAGA NAGLU NPC1 ACP2 ATP6AP1 CTSA PPT1 PSAP MCOLN1 SGSH GNPTAB CLTCL1 GNPTG CTSF AP1G2 AP4M1 SCARB2 LITAF
3.31E-08	50/169	2.5	Path:hsa04141 Protein processing in endoplasmic reticulum	PREB PDIA6 SEC23A OS9 ERP29 EDEM2 SEC63 MAN1B1 TRAM1L1 DDIT3 DDOST SEC31A GANAB TRAM1 DNAJB2 HSPA5 HSPA6 HSPA8 STT3A ATF4 NFE2L2 P4HB DERL2 UBXN1 DNAJB11 UGGT2 SELENOS DNAJC3 ERO1B MAN1C1 BAG1 RAD23A BCL2 RPN1 SEL1L DNAJC1 SIL1 HSP90B1 UFD1 XBP1 TUSC3 UBXN6 CALR SYVN1 RNF185 EIF2AK3 PDIA4 EDEM1 HERPUD1 SEC24D

Disease Models & Mechanisms • Supplementary information

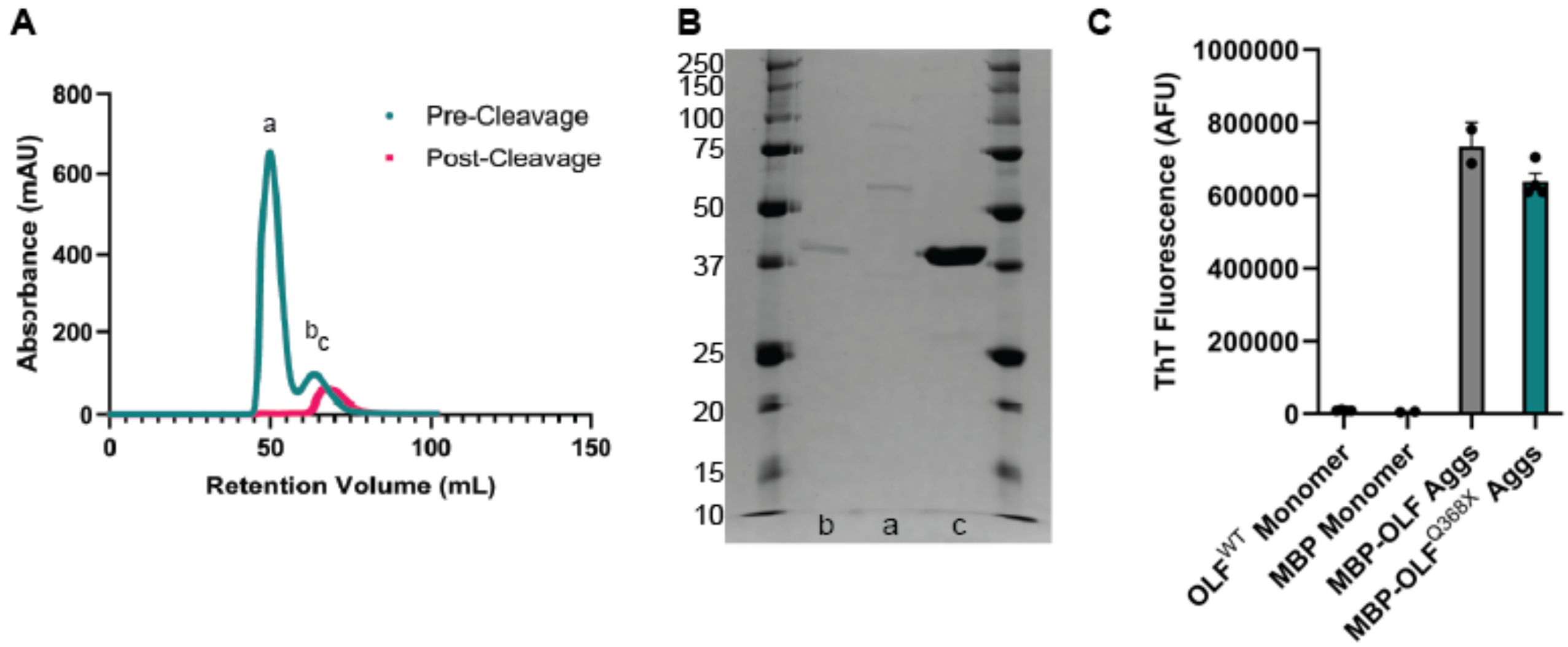

Fig. S1. MBP-OLF^{Q368X} is purified in a misfolded state. (**A**) Size exclusion profile of OLF^{Q368X} prior to TEV cleavage of the MPB tag (teal, peaks a and b) and after cleavage (red, peak c). Exclusion volume ~50 ml. (**B**) Coomassie stained gel of peaks a, b, and c. The species at ~40 kDa is MBP and the species at ~55 kDa is MBP-TEV-OLF^{Q368X}. Post cleavage, OLF^{Q368X} aggregates and precipitates out of solution (not shown). (**C**) ThT fluorescence of MBP-OLF^{Q368X} aggregates compared to OLF^{WT} and MBP (n=2-4). Error bars represent SD.

Disease Models & Mechanisms • Supplementary information

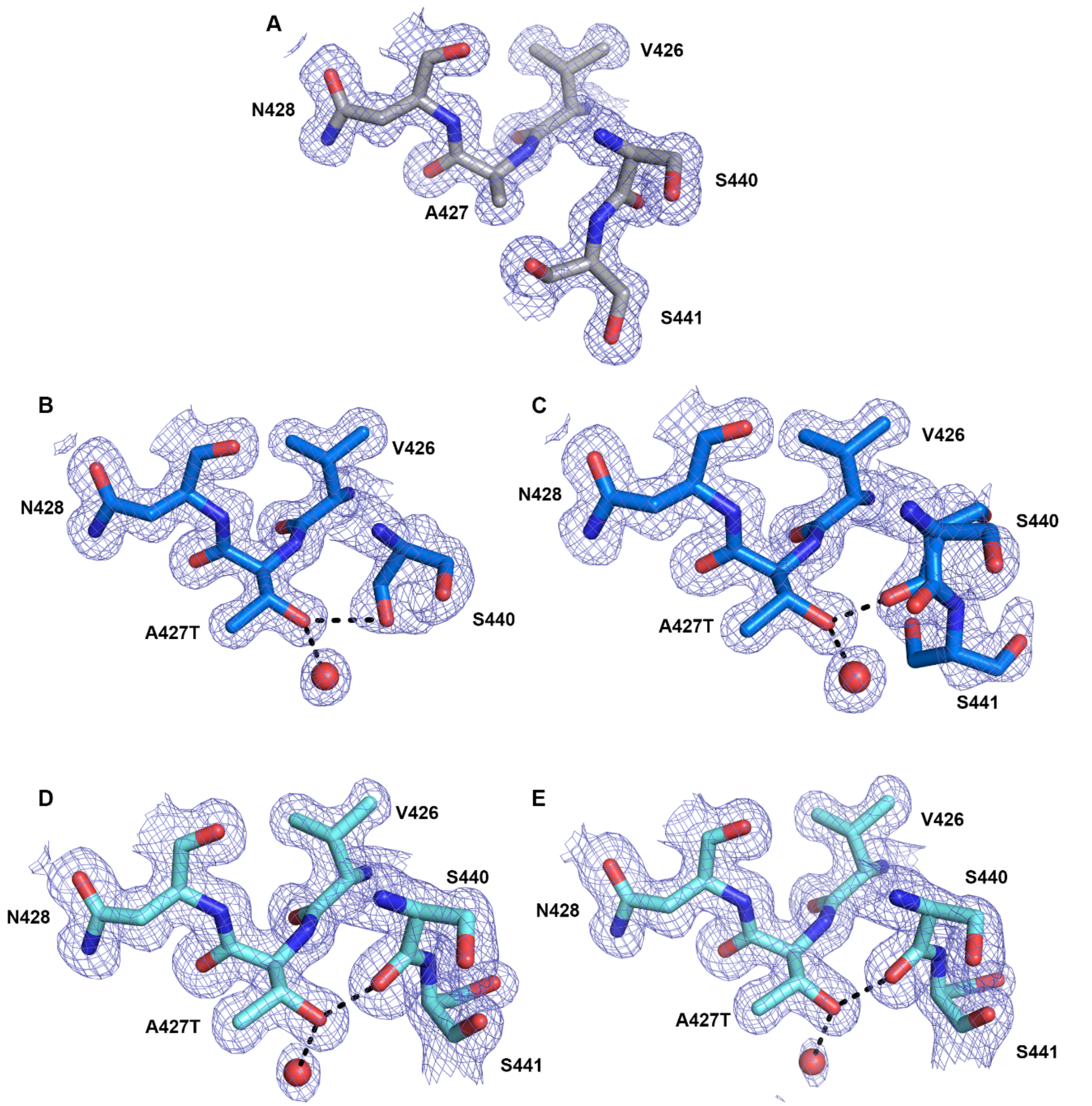

Fig. S2. Interactions formed by A427 in the structure of OLF^{WT} and T427 in the structure of OLF^{A427T}. Zoom into the hydrogen bonding interactions formed by 427 in (A) OLF^{WT} (PDB code 8FRR) compared to OLF^{A427T} (B) chain A, (C) chain B, (D) chain C, and (E) chain D. Black dashed lines indicate interactions within 3 Å of the side chain. 2Fo-2Fc electron density map mesh is contoured at 1σ. Red spheres represent solvent.

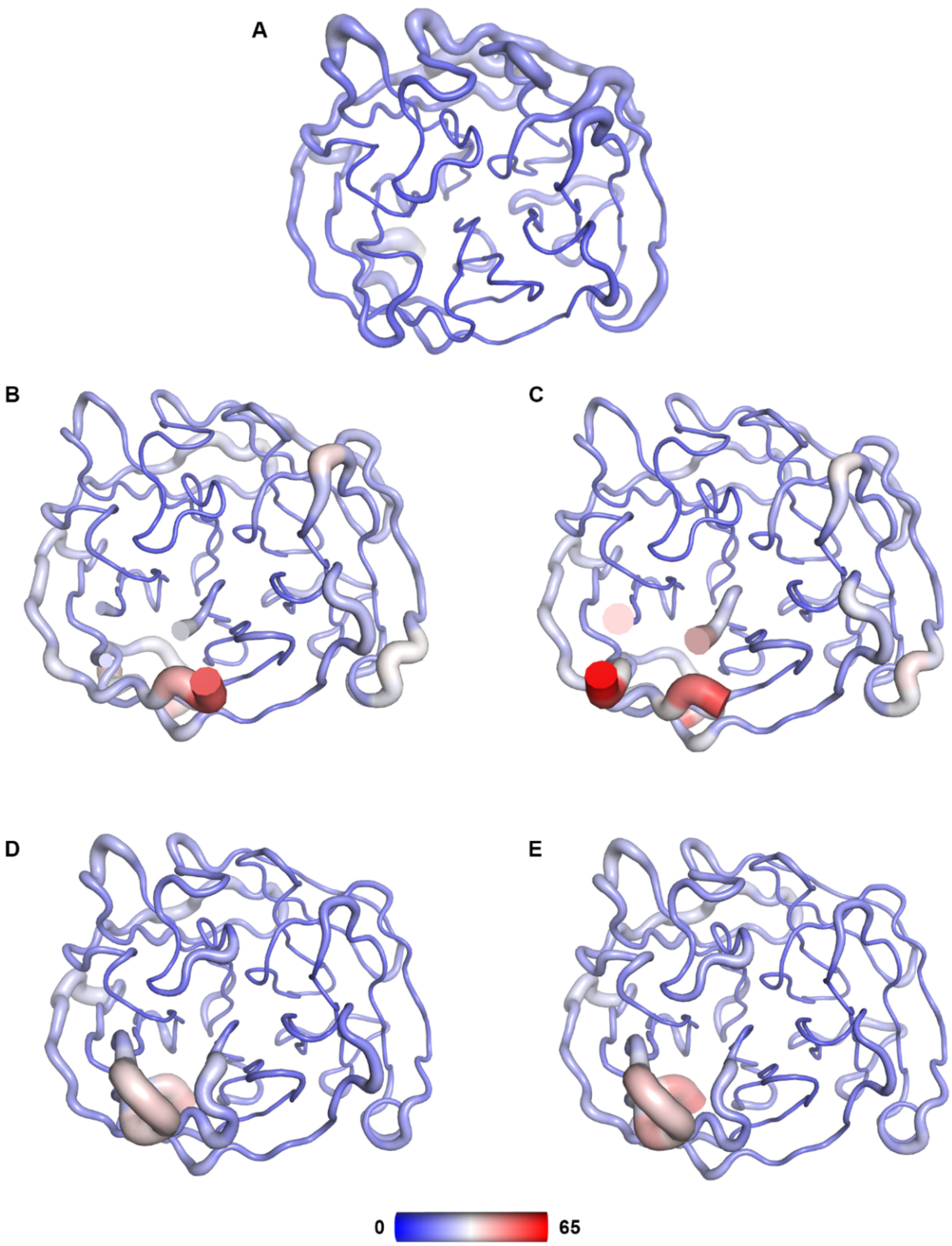

Fig. S3. *Thermal B-factor analysis of OLF^{WT} compared to OLF^{A427T}.* B-factor putty representation of (A) OLF^{WT} (PDB code 8FRR) compared to OLF^{A427T} (B) chain A, (C) chain B, (D) chain C, and (E) chain D. Structure is colored according to B-factor from blue to white to red, from 0 – 65 Å^2.

Disease Models & Mechanisms • Supplementary information

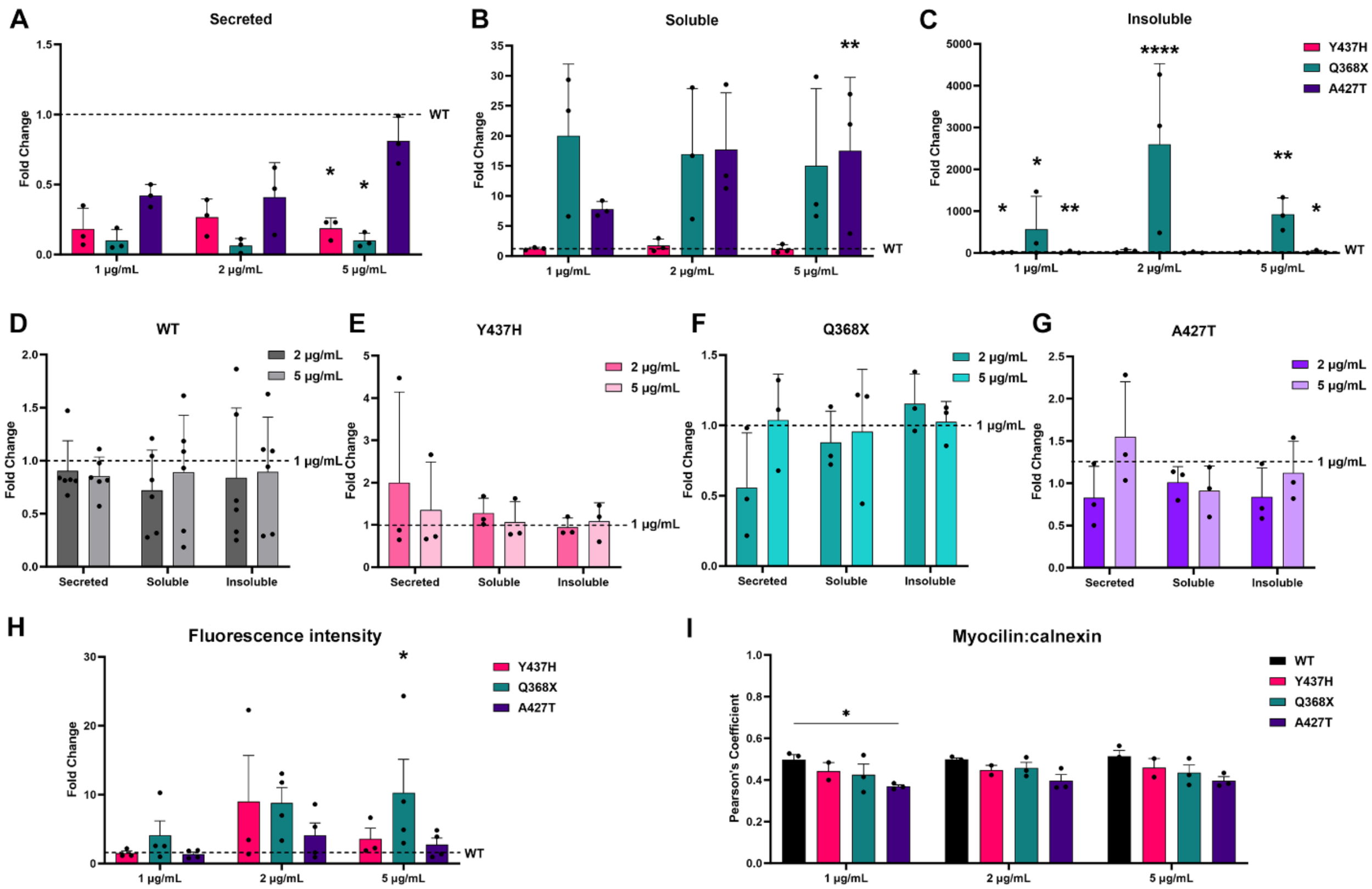

Fig. S4. *Quantification of data shown in Figure 2.* (**A-C**) Quantification of **Figure 2A** where data are the mean fold change of background-subtracted, mean grey values relative to myocilin^{WT} (dotted line) at each doxycycline dose (n=3). β-actin serves as a load control for the detergent-soluble fraction. (**D-G**) Quantification of **Figure 2A** where data are the mean fold change of background-subtracted, mean grey values relative to the 1 µg/mL doxycycline treatment (dotted line) for each variant and fraction, n=3. β-actin serves as a load control for the detergent-soluble fraction. Only significance between doxycycline doses within fractions is shown. (**H**) Quantification of **Figure 2B** where data are mean fold change of background-subtracted, Hoescht-normalized, Triangle-thresholded fluorescence intensity relative to myocilin^{WT} (dotted line) at each doxycycline dose, n=3-4. Data were analyzed by two-way repeated measures ANOVA: *p < 0.05. (**I**) Pearson's correlation coefficient for myocilin and calnexin colocalization in **Figure 2B** from Triangle-thresholded fluorescence intensity. Because Y437H was only stained for calnexin twice, it was excluded from the analysis. Error bars represent standard deviation for A-G and s.e.m. for H-I. Data were analyzed by two-way ANOVA with *, p <0.05; **, p<0.01; ***, p<0.001; ****, p<0.0001.

Disease Models & Mechanisms • Supplementary information

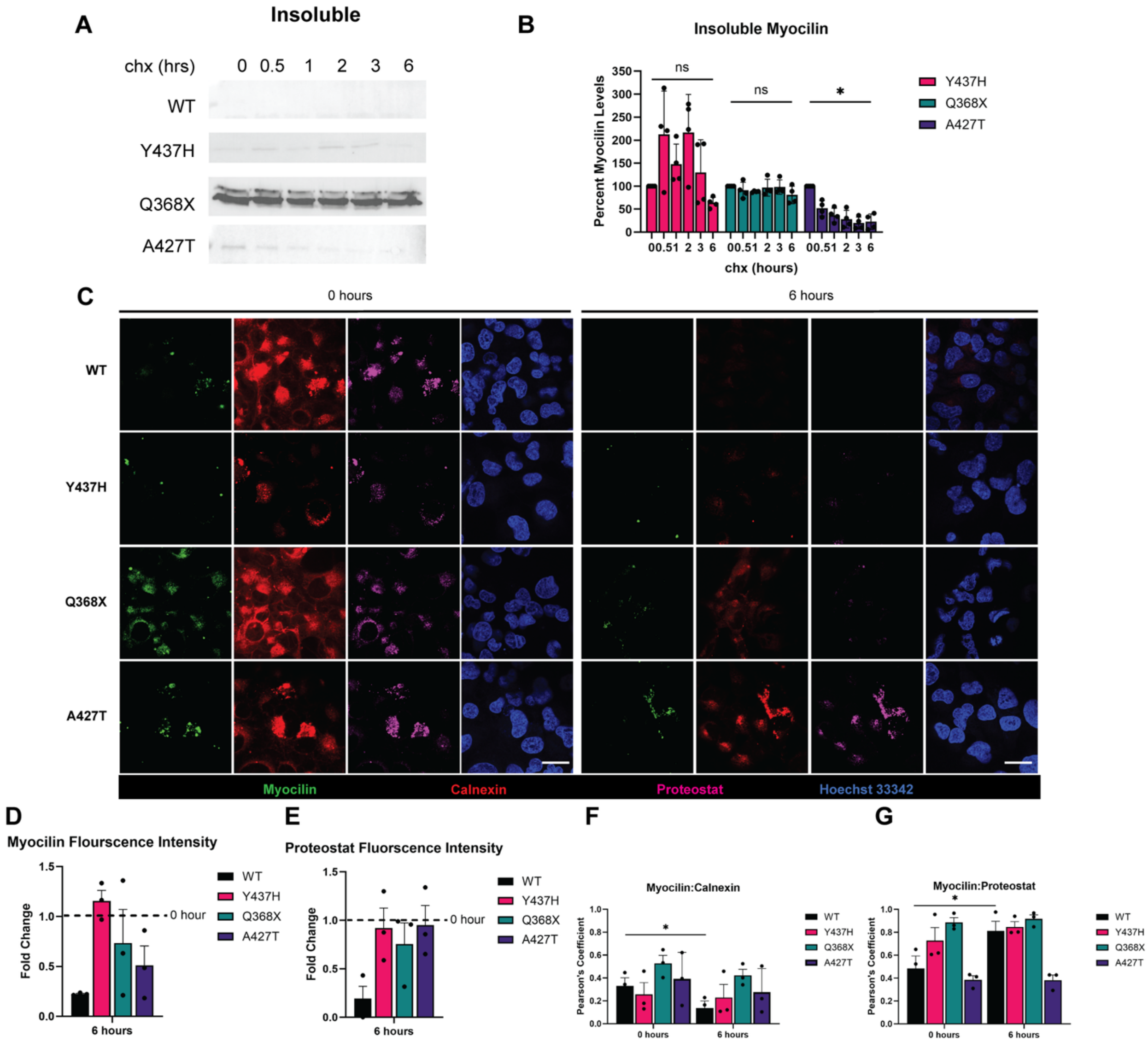

Fig. S5. *Time dependent degradation of myocilin variants after treatment with cycloheximide.* (**A**) Immunoblotting of detergent-insoluble (n=3-4) fractions of myocilin^{WT}, myocilin^{Y437H}, myocilin^{Q368X}, or myocilin^{A427T} induced (2 µg/mL) and treated with cycloheximide (100 µg/mL) for 6 hours. (**B**) Quantification of (A) percent myocilin relative t=0 hours for each (**C**) Immunocytochemistry of myocilin^{WT} and variants induced and treated with cycloheximide (n=3,2). myocilin (green), calnexin (red), Proteostat (magenta), and nuclei (blue). Scale bar is 20 µm. (**D**) Quantification of myocilin fluorescence in (C) intensity relative to 0-hour (dotted line) normalized to Hoeschst and background-subtracted. (**E**) Quantification of Proteostat fluorescence in (C) intensity relative to 0-hour (dotted line) normalized to Hoeschst and background-subtracted. (**F**) Pearson's correlation coefficient for myocilin and calnexin colocalization in (C). (**G**) Pearson's correlation coefficient for myocilin and Proteostat colocalization in (C). Error bars represent standard deviation. Data were analyzed by two-way ANOVA with *, $p < 0.05$.

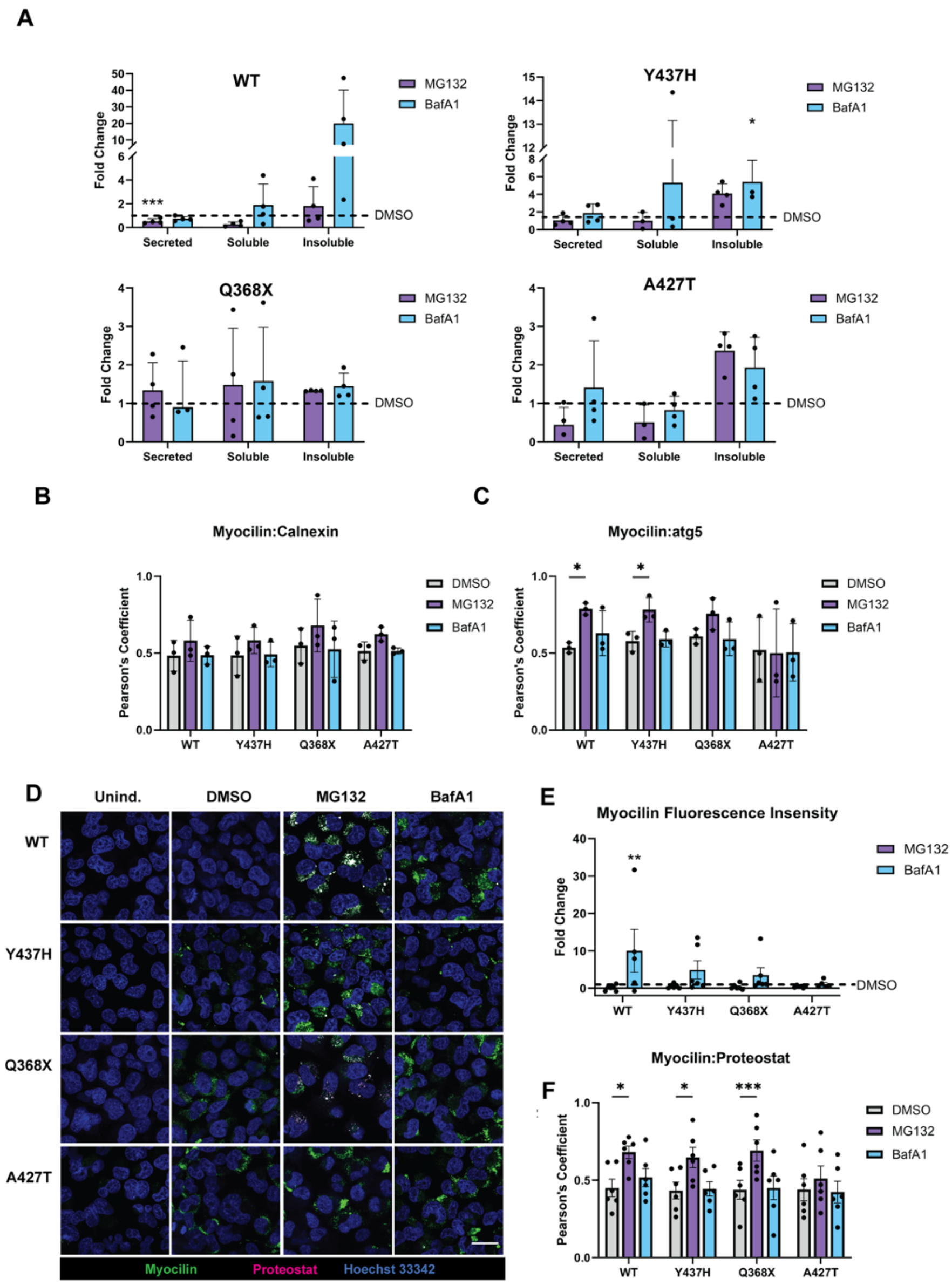

Fig. S6. *Quantification and additional immunofluorescence imaging related to experiments presented in Figure 3.* (**A**) Quantification of **Figure 3A** immunoblotting of secreted, detergent-soluble, and detergent-insoluble of myocilin^{WT}, myocilin^{Y437H}, myocilin^{Q368X}, or myocilin^{A427T} treated with MG132 and BafA1 relative to DMSO vehicle control (dotted line). β-actin serves as a normalization control for the detergent-soluble fraction. (**B**) Pearson's correlation coefficient for myocilin and calnexin colocalization in **Figure 3B**. (**C**) Pearson's correlation coefficient for myocilin and atg5 colocalization in **Figure 3C**. (**D**) Immunocytochemistry of myocilin^{WT} and variants induced and treated with MG132 or BafA1 (n=6). Myocilin (green), Proteostat (magenta), and nuclei (blue). Scale bar is 20 μm. Images are from the same samples as in **Figure 3 B, C**. (**E**) Quantification of myocilin intensity in (D) relative to DMSO (dotted line). (**F**) Pearson's correlation coefficient for myocilin and Proteostat colocalization in (D). Error bars represent standard deviation for A and s.e.m. for B-C, E-F. Data were analyzed by two-way ANOVA *with* *, p <0.05; **, p<0.01; ***, p<0.001.

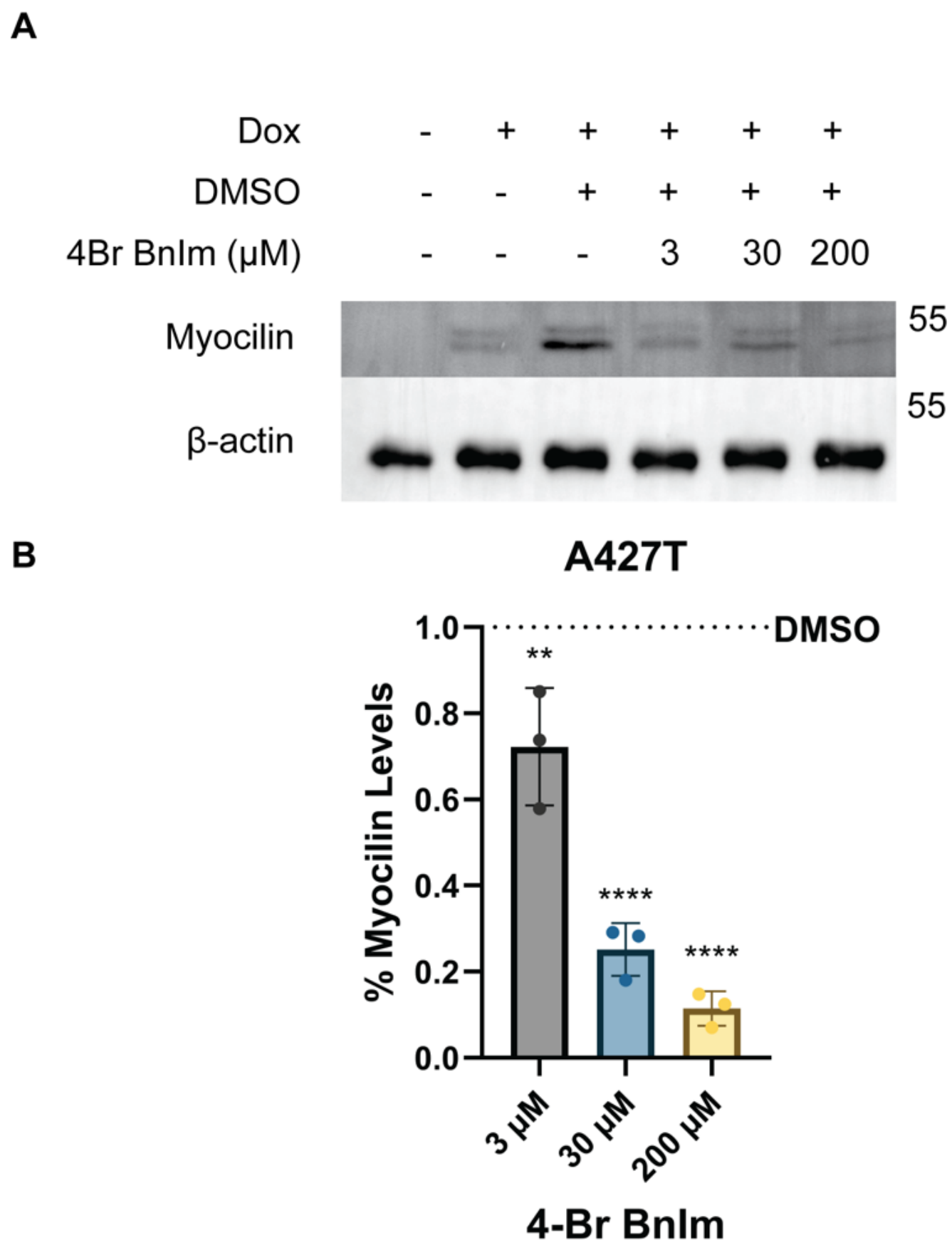

Fig. S7. *Treatment of TM-1 cells expressing myocilin^{A427T} with Grp94 inhibitor 4BrBnIm.* (A) Immunoblotting of detergent-soluble myocilin^{A427T} in transduced TM-1 cells treated with 4-Br-BnIm at concentrations of 3, 30, and 200 μM (n=3) with uninduced controls. β-actin serves as a load control. (B) Quantification of (A) with fold change relative to DMSO treated cells (dotted line) $p > 0.05$; **, $p>0.0001$. Error bars represent standard deviation.

SUPP. REFERENCES

Aldred, M. A., Baumber, L., Hill, A., Schwalbe, E. C., Goh, K., Karwatowski, W. and Trembath, R. C. (2004). Low prevalence of MYOC mutations in UK primary open-angle glaucoma patients limits the utility of genetic testing. *Hum Genet* **115**, 428-431.

Allingham, R. R., Wiggs, J. L., De La Paz, M. A., Vollrath, D., Tallett, D. A., Broomer, B., Jones, K. H., Del Bono, E. A., Kern, J., Patterson, K., et al. (1998). Gln368STOP myocilin mutation in families with late-onset primary open-angle glaucoma. *Invest Ophthalmol Vis Sci* **39**, 2288-2295.

Alward, W. L., Fingert, J. H., Coote, M. A., Johnson, A. T., Lerner, S. F., Junqua, D., Durcan, F. J., McCartney, P. J., Mackey, D. A., Sheffield, V. C., et al. (1998). Clinical features associated with mutations in the chromosome 1 open-angle glaucoma gene (GLC1A). *N Engl J Med* **338**, 1022-1027.

Alward, W. L., Kwon, Y. H., Khanna, C. L., Johnson, A. T., Hayreh, S. S., Zimmerman, M. B., Narkiewicz, J., Andorf, J. L., Moore, P. A., Fingert, J. H., et al. (2002). Variations in the myocilin gene in patients with open-angle glaucoma. *Arch Ophthalmol* **120**, 1189-1197.

Alward, W. L. M., van der Heide, C., Khanna, C. L., Roos, B. R., Sivaprasad, S., Kam, J., Ritch, R., Lotery, A., Igo, R. P., Jr., Cooke Bailey, J. N., et al. (2019). Myocilin Mutations in Patients With Normal-Tension Glaucoma. *JAMA Ophthalmol* **137**, 559-563.

Angius, A., Spinelli, P., Ghilotti, G., Casu, G., Sole, G., Loi, A., Totaro, A., Zelante, L., Gasparini, P., Orzalesi, N., et al. (2000). Myocilin Gln368stop mutation and advanced age as risk factors for late-onset primary open-angle glaucoma. *Arch Ophthalmol* **118**, 674-679.

Baird, P. N., Craig, J. E., Richardson, A. J., Ring, M. A., Sim, P., Stanwix, S., Foote, S. J. and Mackey, D. A. (2003). Analysis of 15 primary open-angle glaucoma families from Australia identifies a founder effect for the Q368STOP mutation of myocilin. *Hum Genet* **112**, 110-116.

Baird, P. N., Foote, S. J., Mackey, D. A., Craig, J., Speed, T. P. and Bureau, A. (2005a). Evidence for a novel glaucoma locus at chromosome 3p21-22. *Hum Genet* **117**, 249-257.

Baird, P. N., Richardson, A. J., Craig, J. E., Rochtchina, E., Mackey, D. A. and Mitchell, P. (2005b). The Q368STOP myocilin mutation in a population-based cohort: the Blue Mountains Eye Study. *Am J Ophthalmol* **139**, 1125-1126.

Baird, P. N., Richardson, A. J., Mackey, D. A., Craig, J. E., Faucher, M. and Raymond, V. (2005c). A common disease haplotype for the Q368STOP mutation of the myocilin gene in Australian and Canadian glaucoma families. *Am J Ophthalmol* **140**, 760-762.

Banerjee, D., Bhattacharjee, A., Ponda, A., Sen, A. and Ray, K. (2012). Comprehensive analysis of myocilin variants in east Indian POAG patients. *Mol Vis* **18**, 1548-1557.

Bennett, S. L., Hewitt, A. W., Poulsen, J. L., Kearns, L. S., Morgan, J. E., Craig, J. E. and Mackey, D. A. (2007). Screening for glaucomatous disc changes prior to diagnosis of glaucoma in myocilin pedigrees. *Arch Ophthalmol* **125**, 112-116.

Bhattacharjee, A., Acharya, M., Mukhopadhyay, A., Mookherjee, S., Banerjee, D., Bandopadhyay, A. K., Thakur, S. K., Sen, A. and Ray, K. (2007). Myocilin variants in Indian patients with open-angle glaucoma. *Arch Ophthalmol* **125**, 823-829.

Cobb, C. J., Scott, G., Swingler, R. J., Wilson, S., Ellis, J., MacEwen, C. J. and McLean, W. H. (2002). Rapid mutation detection by the transgenomic wave analyser DHPLC identifies MYOC mutations in patients with ocular hypertension and/or open angle glaucoma. *Br J Ophthalmol* **86**, 191-195.

Craig, J. E., Baird, P. N., Healey, D. L., McNaught, A. I., McCartney, P. J., Rait, J. L., Dickinson, J. L., Roe, L., Fingert, J. H., Stone, E. M., et al. (2001). Evidence for genetic heterogeneity within eight glaucoma families, with the GLC1A Gln368STOP mutation being an important phenotypic modifier. *Ophthalmology* **108**, 1607-1620.

Ennis, S., Gibson, J., Griffiths, H., Bunyan, D., Cree, A. J., Robinson, D., Self, J., MacLeod, A. and Lotery, A. (2010). Prevalence of myocilin gene mutations in a novel UK cohort of POAG patients. *Eye (Lond)* **24**, 328-333.

Faucher, M., Anctil, J. L., Rodrigue, M. A., Duchesne, A., Bergeron, D., Blondeau, P., Cote, G., Dubois, S., Bergeron, J., Arseneault, R., et al. (2002). Founder TIGR/myocilin mutations for glaucoma in the Quebec population. *Hum Mol Genet* **11**, 2077-2090.

Fingert, J. H., Clark, A. F., Craig, J. E., Alward, W. L., Snibson, G. R., McLaughlin, M., Tuttle, L., Mackey, D. A., Sheffield, V. C. and Stone, E. M. (2001). Evaluation of the myocilin (MYOC) glaucoma gene in monkey and human steroid-induced ocular hypertension. *Invest Ophthalmol Vis Sci* **42**, 145-152.

Fingert, J. H., Heon, E., Liebmann, J. M., Yamamoto, T., Craig, J. E., Rait, J., Kawase, K., Hoh, S. T., Buys, Y. M., Dickinson, J., et al. (1999). Analysis of myocilin mutations in 1703 glaucoma patients from five different populations. *Hum Mol Genet* **8**, 899-905.

Graul, T. A., Kwon, Y. H., Zimmerman, M. B., Kim, C. S., Sheffield, V. C., Stone, E. M. and Alward, W. L. (2002). A case-control comparison of the clinical characteristics of glaucoma and ocular hypertensive patients with and without the myocilin Gln368Stop mutation. *Am J Ophthalmol* **134**, 884-890.

Hewitt, A. W., Bennett, S. L., Dimasi, D. P., Craig, J. E. and Mackey, D. A. (2006). A myocilin Gln368STOP homozygote does not exhibit a more severe glaucoma phenotype than heterozygous cases. *Am J Ophthalmol* **141**, 402-403.

Hogewind, B. F., Mukhopadhyay, A., Theelen, T., Hollander, A. I. and Hoyng, C. B. (2010). Variable clinical spectrum of the myocilin Gln368X mutation in a Dutch family with primary open angle glaucoma. *Curr Eye Res* **35**, 31-36.

Jansson, M., Marknell, T., Tomic, L., Larsson, L. I. and Wadelius, C. (2003). Allelic variants in the MYOC/TIGR gene in patients with primary open-angle, exfoliative glaucoma and unaffected controls. *Ophthalmic Genet* **24**, 103-110.

Liu, T., Tang, C. and Shi, X. (2020). Analysis of variants in Chinese individuals with primary open-angle glaucoma using molecular inversion probe (MIP)-based panel sequencing. *Mol Vis* **26**, 378-391.

Liu, W., Liu, Y., Challa, P., Herndon, L. W., Wiggs, J. L., Girkin, C. A., Allingham, R. R. and Hauser, M. A. (2012). Low prevalence of myocilin mutations in an African American population with primary open-angle glaucoma. *Mol Vis* **18**, 2241-2246.

Liuska, P. J., Tadji, A., Repo, P., Hiltunen, J., Backlund, M., Jarvinen, R. S., Ojanen, E., Majander, A., Kivela, T. T., Harju, M., et al. (2023). Analysis of glaucoma genes in Finnish patients with juvenile open-angle glaucoma. *Acta Ophthalmol* **101**, 797-806.

Lopez-Martinez, F., Lopez-Garrido, M. P., Sanchez-Sanchez, F., Campos-Mollo, E., Coca-Prados, M. and Escribano, J. (2007). Role of MYOC and OPTN sequence variations in Spanish patients with primary open-angle glaucoma. *Mol Vis* **13**, 862-872.

Mardin, C. Y., Velten, I., Ozbey, S., Rautenstrauss, B. and Michels-Rautenstrauss, K. (1999). A GLC1A gene Gln368Stop mutation in a patient with normal-tension open-angle glaucoma. *J Glaucoma* **8**, 154-156.

Mataftsi, A., Achache, F., Heon, E., Mermoud, A., Cousin, P., Metthez, G., Schorderet, D. F. and Munier, F. L. (2001). MYOC mutation frequency in primary open-angle glaucoma patients from Western Switzerland. *Ophthalmic Genet* **22**, 225-231.

Melki, R., Belmouden, A., Brézin, A. and Garchon, H.-J. (2003). Myocilin analysis by DHPLC in French POAG patients: Increased prevalence of Q368X mutation. *Human Mutation* **22**, 179-179.

Michels-Rautenstrauss, K., Mardin, C., Wakili, N., Junemann, A. M., Villalobos, L., Mejia, C., Soley, G. C., Azofeifa, J., Ozbey, S., Naumann, G. O., et al. (2002). Novel mutations in the MYOC/GLC1A gene in a large group of glaucoma patients. *Hum Mutat* **20**, 479-480.

Milla, E., Mane, B., Duch, S., Hernan, I., Borras, E., Planas, E., Dias Mde, S., Carballo, M., Gamundi, M. J. and Spanish Multicenter Glaucoma Group-Estudio Multicentrico Espanol de Investigacion Genetica del Glaucoma, E. (2013). Survey of familial glaucoma shows a high incidence of cytochrome P450, family 1, subfamily B, polypeptide 1 (CYP1B1) mutations in non-consanguineous congenital forms in a Spanish population. *Mol Vis* **19**, 1707-1722.

O'Gorman, L., Cree, A. J., Ward, D., Griffiths, H. L., Sood, R., Denniston, A. K., Self, J. E., Ennis, S., Lotery, A. J. and Gibson, J. (2019). Comprehensive sequencing of the myocilin gene in a selected cohort of severe primary open-angle glaucoma patients. *Sci Rep* **9**, 3100.

Rakhmanov, V. V., Nikitina, N., Zakharova, F. M., Astakhov Iu, S., Kvasova, M. D., Vasil'ev, V. B., Golubkov, V. I. and Mandel'shtam, M. (2005). [Mutations and polymorphisms in the genes for myocilin and optineur in as the risk factors of primary open-angle glaucoma]. *Genetika* **41**, 1567-1574.

Sherwin, J. C., Hewitt, A. W., Bennett, S. L., Baird, P. N., Craig, J. E. and Mackey, D. A. (2009). Primary open angle glaucoma in subjects harbouring the predicted GLC1L haplotype reveals a normotensive phenotype. *Clin Exp Ophthalmol* **37**, 201-207.

Shimizu, S., Lichter, P. R., Johnson, A. T., Zhou, Z., Higashi, M., Gottfredsdottir, M., Othman, M., Moroi, S. E., Rozsa, F. W., Schertzer, R. M., et al. (2000). Age-dependent prevalence of mutations at the GLC1A locus in primary open-angle glaucoma. *Am J Ophthalmol* **130**, 165-177.

Souzeau, E., Burdon, K. P., Dubowsky, A., Grist, S., Usher, B., Fitzgerald, J. T., Crawford, A., Hewitt, A. W., Goldberg, I., Mills, R. A., et al. (2013). Higher prevalence of myocilin mutations in advanced glaucoma in comparison with less advanced disease in an Australasian disease registry. *Ophthalmology* **120**, 1135-1143.

Stone, E. M., Fingert, J. H., Alward, W. L., Nguyen, T. D., Polansky, J. R., Sunden, S. L., Nishimura, D., Clark, A. F., Nystuen, A., Nichols, B. E., et al. (1997). Identification of a gene that causes primary open angle glaucoma. *Science* **275**, 668-670.

Svidnicki, P. V., Braghini, C. A., Costa, V. P., Schimiti, R. B., de Vasconcellos, J. P. C. and de Melo, M. B. (2018). Occurrence of MYOC and CYP1B1 variants in juvenile open angle glaucoma Brazilian patients. *Ophthalmic Genet* **39**, 717-724.

Vazquez, C. M., Herrero, O. M., Bastus, B. M. and Perez, V. D. (2000). Mutations in the third exon of the MYOC gene in spanish patients with primary open angle glaucoma. *Ophthalmic Genet* **21**, 109-115.

Villalba, M. F., Grajewski, A. L., Tekin, M., Bademci, G. and Chang, T. C. (2022). Diagnostic yield of next generation sequencing gene panel assays for early-onset glaucoma in an ethnically diverse population. *J AAPOS* **26**, 302 e301-302 e306.

Vincent, A. L., Billingsley, G., Buys, Y., Levin, A. V., Priston, M., Trope, G., Williams-Lyn, D. and Heon, E. (2002). Digenic inheritance of early-onset glaucoma: CYP1B1, a potential modifier gene. *Am J Hum Genet* **70**, 448-460.

Wiggs, J. L., Allingham, R. R., Vollrath, D., Jones, K. H., De La Paz, M., Kern, J., Patterson, K., Babb, V. L., Del Bono, E. A., Broomer, B. W., et al. (1998). Prevalence of mutations in TIGR/Myocilin in patients with adult and juvenile primary open-angle glaucoma. *Am J Hum Genet* **63**, 1549-1552.

Wiggs, J. L., Howell, G. R., Linkroum, K., Abdrabou, W., Hodges, E., Braine, C. E., Pasquale, L. R., Hannon, G. J., Haines, J. L. and John, S. W. (2013). Variations in COL15A1 and COL18A1 influence age of onset of primary open angle glaucoma. *Clin Genet* **84**, 167-174.

Williams-Lyn, D., Flanagan, J., Buys, Y., Trope, G. E., Fingert, J., Stone, E. M. and Heon, E. (2000). The genetic aspects of adult-onset glaucoma: a perspective from the Greater Toronto area. *Can J Ophthalmol* **35**, 12-17.

Willoughby, C. E., Chan, L. L., Herd, S., Billingsley, G., Noordeh, N., Levin, A. V., Buys, Y., Trope, G., Sarfarazi, M. and Heon, E. (2004). Defining the pathogenicity of optineurin in juvenile open-angle glaucoma. *Invest Ophthalmol Vis Sci* **45**, 3122-3130.

Young, T. K., Souzeau, E., Liu, L., Kearns, L. S., Burdon, K. P., Craig, J. E. and Ruddle, J. B. (2012). Compound heterozygote myocilin mutations in a pedigree with high prevalence of primary open-angle glaucoma. *Mol Vis* **18**, 3064-3069.
